# Myriad factors and pathways influencing tumor radiotherapy resistance

**DOI:** 10.1515/biol-2022-0992

**Published:** 2024-11-26

**Authors:** Lanjuan Mi, Hongquan Zhang

**Affiliations:** School of Life and Health Sciences, Huzhou College, Hu Zhou, China; The First Affiliated Hospital of Huzhou University, Hu Zhou, China

**Keywords:** radiotherapy, hypoxia, tumor microenvironment, therapeutic resistance

## Abstract

Radiotherapy is a cornerstone in the treatment of various tumors, yet radioresistance often leads to treatment failure and tumor recurrence. Several factors contribute to this resistance, including hypoxia, DNA repair mechanisms, and cancer stem cells. This review explores the diverse elements that drive tumor radiotherapy resistance. Historically, resistance has been attributed to cellular repair and tumor repopulation, but recent research has expanded this understanding. The tumor microenvironment – characterized by hypoxia, immune evasion, and stromal interactions – further complicates treatment. Additionally, molecular mechanisms such as aberrant signaling pathways, epigenetic modifications, and non-B-DNA structures play significant roles in mediating resistance. This review synthesizes current knowledge, highlighting the interplay of these factors and their clinical implications. Understanding these mechanisms is crucial for developing strategies to overcome resistance and improve therapeutic outcomes in cancer patients.

## 1 Introduction

As cancer incidence and mortality rise globally, this disease poses a serious threat to human health, reducing life expectancy. According to the latest global cancer statistics from the World Health Organization and the International Agency for Research on Cancer, 19.3 million new cancer cases were reported in 2020, leading to nearly 10 million deaths. By 2040, cancer-related deaths could surge to 28.4 million [1,2]. In response, various strategies have been developed, including surgery, chemotherapy, radiotherapy, immunotherapy, and more [3]. Among these, radiotherapy has been a critical treatment modality since Marie Curie’s discovery of radioactivity [4,5].

**Figure 1:**
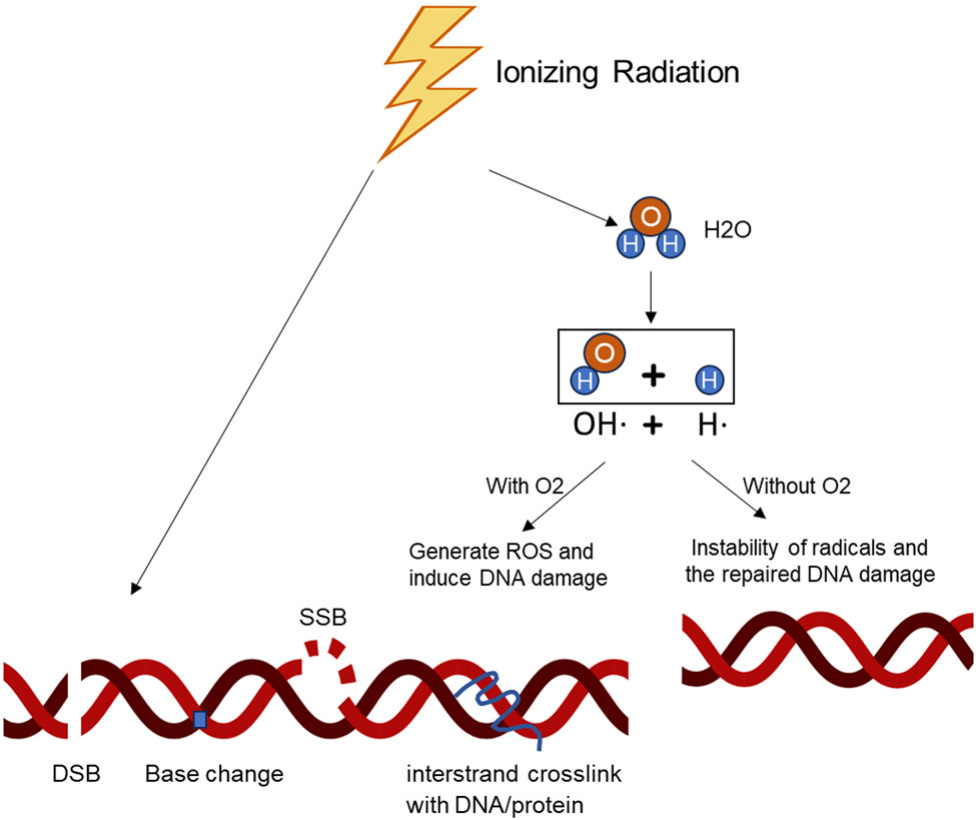
Ionizing radiation (IR) mechanism in radiotherapy: IR directly damages DNA, leading to potential cell death or permanent growth arrest if the damage is not repaired. Indirectly, IR generates ROS through the radiolysis of water and oxygen in normoxic conditions, causing additional DNA damage. Under hypoxic conditions, ROS generation is reduced, diminishing DNA damage.

The primary goal of ionizing radiotherapy is to control localized tumors by inducing DNA damage and apoptosis in cancer cells. High-energy photon radiation, such as X-rays and γ-rays, is typically used. Radiotherapy works through both direct and indirect mechanisms: it directly causes single-strand breaks (SSB) and double-strand breaks (DSB) in DNA, halting cell proliferation and leading to cell death, while indirectly producing reactive oxygen species (ROS) that amplify DNA damage in normoxic tissues (Figure 1). Technological advancements, such as intensity-modulated radiotherapy, stereotactic body radiotherapy (SBRT), image-guided radiotherapy, and proton therapy, have optimized radiation delivery and minimized collateral damage. However, challenges remain, particularly the enhanced DNA damage response (DDR) and tumor heterogeneity, which hinder the efficacy of radiotherapy as a stand-alone treatment [6,7,8]. Therefore, understanding the mechanisms behind tumor radiotherapy resistance is crucial.

This article focuses on the cellular and molecular factors contributing to tumor radiotherapy resistance and outlines future research directions based on these factors.

## 2 DNA repair mechanisms and cell cycle

### 2.1 Overview of DNA damage and repair

Radiotherapy exerts its therapeutic effect primarily through the cytotoxic DNA damage it induces in proliferating cancer cells. This damage occurs either through direct absorption of radiation energy or indirectly via free radicals produced by IR [9]. DNA damage includes base damage, interstrand crosslinks, SSB, and, most notably, DSB, the latter being the most harmful [10] (Figure 1). In response, tumor cells initiate the DDR and arrest the cell cycle to repair the damaged DNA. Different types of DNA damage activate various repair mechanisms, such as mismatch repair, nucleotide excision repair (NER), non-homologous end joining (NHEJ), and homologous recombination (HR) for DSBs [11]. Simultaneously, DNA damage checkpoints are activated, halting the cell cycle to allow for repair. The efficiency of the DDR is crucial in determining the fate of cancer cells following IR.

During cancer cell evolution, several molecular signaling pathways have emerged to counteract radiation-induced damage, contributing to cancer cell radioresistance and, ultimately, radiotherapy failure [12]. Furthermore, a subset of cancer cells not only becomes more radioresistant but also more aggressive, promoting metastasis [13]. Therefore, understanding the factors that enhance DDR in tumor cells is critical for improving their radiosensitivity.

### 2.2 Enhanced DDR leads to radioresistance

DNA damage activates a cascade of biochemical reactions in response to IR, triggering various cellular responses. DNA damage sensors detect the damage and recruit DDR core kinases and other regulatory proteins to the damage sites, initiating the repair process [14,15].

#### 2.2.1 DNA damage sensors

**Figure 2:**
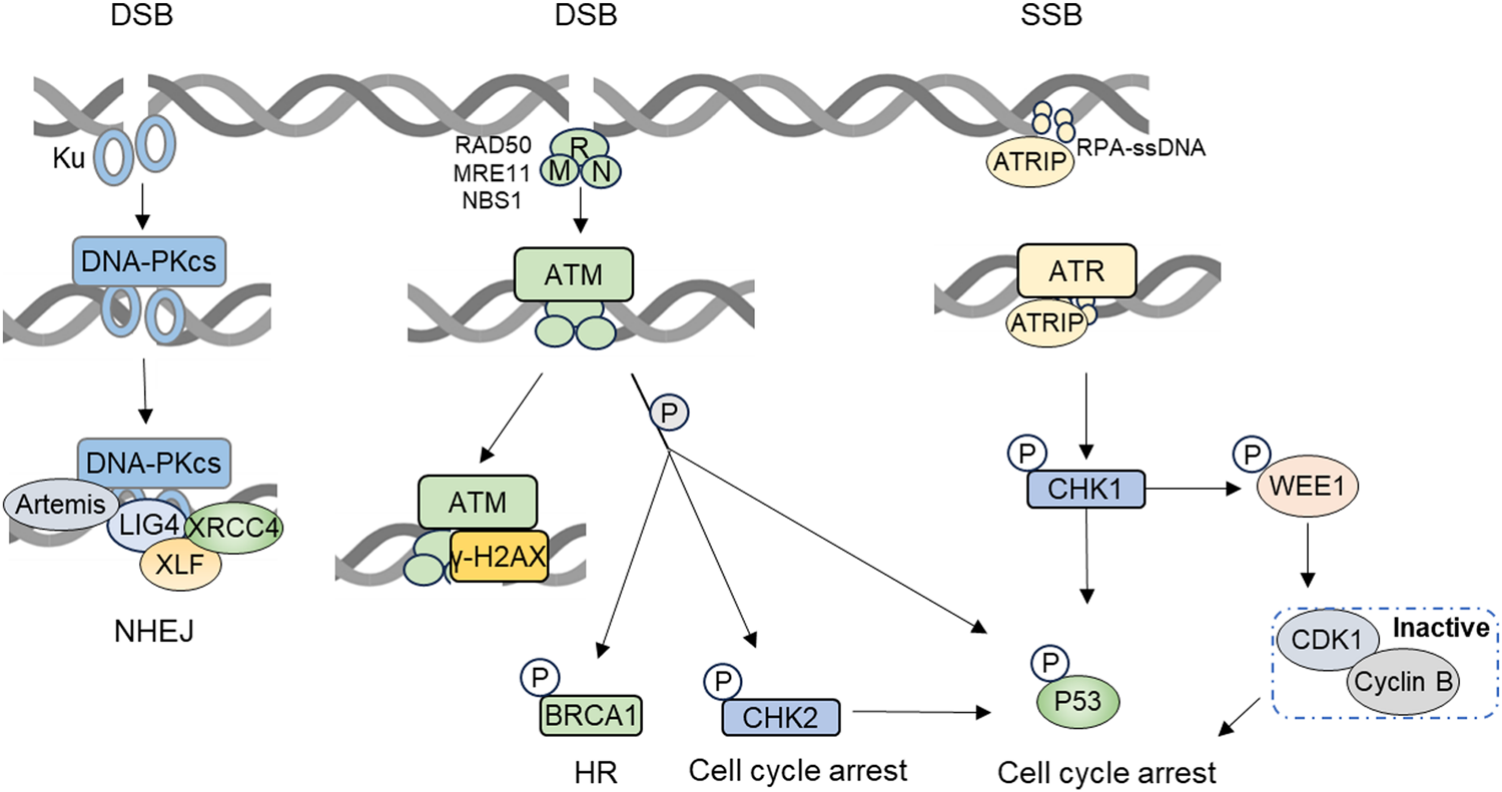
DNA-PKcs are mobilized and activated by the Ku-bound DSB termini. The MRN complex facilitates ATM recruitment to DSBs. ATR binds to replication protein A (RPA)-coated ssDNA through ATRIP, initiating various pathways responsible for DNA repair and cell cycle arrest post-radiation.

Sensors such as H2AX, the MRE11-RAD50-NBS1 (MRN) complex, Ku70/Ku80, MDC1, and 53BP1 recognize DNA damage signals. They recruit DDR core kinases and regulatory elements to the DNA break sites, initiating the repair process (Figure 2) [16,17,18]. Notably, H2AX is rapidly phosphorylated in response to DSBs, forming γH2AX foci, which serve as markers for DSBs and provide insights into the effectiveness of radiotherapy [19]. The MRN complex plays a key role in DSB sensing and signal transduction, initiating repair pathways [20]. Ho et al. identified that elevated expression of the MRN complex has been linked to increased radioresistance and poor outcomes in rectal cancer [21]. Similarly, high expression levels of Ku70/Ku80, MDC1, and 53BP1 are associated with radioresistance across multiple cancers [22,23,24]. Thus, these sensors may serve as reliable markers for predicting clinical radiotherapy outcomes.

#### 2.2.2 DDR core kinases

DDR core kinases, including DNA-dependent protein kinase catalytic subunit (DNA-PKcs), ataxia-telangiectasia mutated (ATM), and ATM and Rad3-related (ATR), initiate the DDR signaling cascade in mammalian cells [17]. DNA-PK, comprising Ku70, Ku80, and DNA-PKcs, is essential for the NHEJ pathway, responding quickly to DSBs. Ku70/80 senses DSBs and initiates NHEJ, followed by the recruitment of DNA-PKcs to the damaged site [25]. DNA-PKcs undergoes self-phosphorylation and phosphorylates downstream targets [17,26]. A suppressor of DNA repair, LRRC31, interacts with Ku70/Ku80 and ATR, inhibiting DNA-PKcs recruitment and enhancing the radiosensitivity of breast cancer brain metastasis to radiation [27]. DNA-PKcs also stabilize SOX2 in glioma stem-like cells (GSCs), promoting resistance by maintaining GSCs in a stem cell state. Inhibiting DNA-PKcs pharmacologically enhances the radiosensitivity of glioblastoma xenografts [28]. Furthermore, recent studies have shown that long non-coding RNAs (lncRNA) LINC00312 [29] and microRNA (miRNA) miR-145 [30,31] hinder DNA-PKcs recruitment to DSB sites, promoting degradation and increasing tumor radiosensitivity.

When DNA damage occurs, ATM and ATR activate DDR repair mechanisms, promoting either cell survival or apoptosis (Figure 2). ATM is vital for maintaining cell cycle checkpoints, repairing IR-induced DSBs, and telomere maintenance [32]. In response to DSBs, the MRN complex recruits ATM, which activates the DDR cascade, including γH2AX signaling and phosphorylation of substrates such as checkpoint kinase 2 (CHK2), leading to cell cycle arrest and allowing time for DNA repair [17]. ATR is activated by single-stranded DNA (ssDNA) and, through ATR-interacting protein (ATRIP), binds to ssDNA at damage sites [33]. ATR then phosphorylates CHK1, causing a cell-cycle arrest at the G2-M phase to facilitate DNA repair [17]. Enhanced activation of ATR-CHK1 and ATM-CHK2 pathways has been reported in cancer stem cells (CSCs) of several solid tumors, contributing to their radioresistance [34,35,36]. Increased ATM or ATR expression in tumors is also associated with radiation resistance [37,38].

Given their key roles in DDR, ATM and ATR kinases are promising targets for improving radiotherapy outcomes. Reported ATM inhibitors include caffeine, wortmannin, CP-466722, KU-55933, KU-60019, and KU-559403, while ATR inhibitors include schisandrin B, NU6027, NVP-BEZ235, VE-821, VE-822, AZ20, and AZD6738 [39,40]. However, concerns about increased toxicity to normal tissues remain, and further examination is required to optimize their selective use alongside radiotherapy.

#### 2.2.3 Other DDR proteins

Poly(ADP-ribose) polymerase (PARP), a nuclear enzyme, is one of the 18 members of the PARP protein family. The PARP family senses DNA gaps, transfers single ADP-ribose or PAR to itself or other proteins, and recruits DNA repair proteins to DNA damage sites, playing a pivotal role in DNA repair [41,42]. Among them, PARP-1, a prominent member, is the most extensively researched nuclear enzyme in relation to DNA repair [43,44]. Specifically, PARP-1 is involved in base excision repair following SSBs [43] and participates in alternative DNA repair processes such as NER, enhancing cancer cell resistance to radiation [45]. While inhibiting PARP-1 compromises its repair capabilities for DNA SSB, this inhibition is not lethal since DNA damage can be rectified through other pathways, predominantly HR [46]. Notably, breast-cancer-associated genes 1 and 2 (BRCA1 and BRCA2) are vital components of HR [47,48]. Mutations in these genes, when combined with PARP-1 inactivation, lead to synthetic lethality and cell death [49,50]. To enhance the radiosensitivity of cancers with BRCA1/2 mutations, such as prostate, breast, and pancreatic cancers, clinicians frequently employ IR alongside PARP inhibitors [51,52,53,54]. A clinical trial focusing on triple-negative breast cancer (TNBC) demonstrated that combining the PARP inhibitor olaparib with radiotherapy at an early stage increased radiotherapy efficacy and exhibited promising safety profiles for high-risk TNBC patients [55].

In addition to DNA damage sensors and kinases, cell cycle checkpoint kinases such as CHK1 and G2 Checkpoint Kinase not only provide time for DNA repair but also contribute to tumor radiotherapy resistance [56,57]. As shown in Figure 2, ssDNA activates ATR, which phosphorylates and activates CHK1. CHK1 then phosphorylates CDC25C and WEE1, leading to the activation of WEE1 and the inactivation of CDC25C. This results in G2 phase arrest, allowing DNA repair [58,59]. CHK1 and WEE1 exhibit heightened expression in several cancer types, notably high-grade serous ovarian carcinoma and breast cancer [60,61]. Due to their roles in DDR, CHK1 and WEE1 have been targeted as anticancer agents. Inhibiting WEE1, CHK1, or both prompts tumor cells to proceed into mitosis without repairing DNA, triggering apoptosis or cell death. This approach has shown promise clinically, especially in ovarian cancer. However, early-stage clinical trials have shown that the first-in-human WEE1 inhibitor, adavosertib, is limited by dose-limiting adverse events [59]. Thus, combining WEE1 inhibitors with radiotherapy may alleviate adverse effects. Additionally, studies have found that post-radiation, tumor cells recruit caspase-activated DNase (CAD) to DNA damage sites, selectively cleaving DNA and inhibiting replication. Inhibition of CAD contributes to radiotherapy sensitivity [62]. Thus, targeting the G2 cycle checkpoint may improve radiotherapy efficacy.

To enhance radiotherapy sensitivity and improve survival rates, researchers are focusing on small molecular inhibitors targeting DNA repair pathways and cell cycle checkpoint kinases. Among emerging therapies, PARP inhibitors have been particularly successful, exploiting synthetic lethality to target tumors. Several PARP inhibitors, such as Talazoparib, Rucaparib, Niraparib, and Olaparib, have been approved by the food and drug administration (FDA) for oncological use [63]. However, resistance to DDR inhibitors remains challenging in clinical settings, leading to increased recurrence and diminished outcomes. Resistance mechanisms include reversion mutations, epigenetic changes, replication fork stabilization, and increased drug efflux [64,65]. Understanding these mechanisms and developing strategies to counter resistance is crucial for optimizing radiotherapy outcomes.

## 3 Tumor microenvironment (TME) and radiotherapy resistance

In oncology, the understanding of cancer has expanded beyond a cancer cell-centric view to encompass non-malignant cells and other non-cellular components, collectively known as the TME. The TME includes tumor cells, infiltrating immune cells, cancer-associated fibroblasts (CAFs), their secretions, extracellular matrix (ECM) components, and hypoxia [66]. TME studies, including preclinical tumor models, have shown that TME cells and their secretions play a key role in cancer pathogenesis and therapeutic resistance, making them attractive therapeutic targets.

### 3.1 Hypoxia

#### 3.1.1 Role of tumor hypoxia in therapy resistance

Tumor hypoxia is a common feature of the microenvironment in solid tumors [67,68], primarily resulting from an imbalance between poor vascularization and high oxygen consumption by tumor cells [69]. Hypoxia induces a tumor-adapted phenotype, altering signaling, gene expression, and metabolism [70]. Both hypoxia and hypoxia-induced phenotypic changes contribute to radiotherapy resistance [71], making hypoxia a critical predictor of treatment resistance and poor clinical outcomes [72]. Understanding the factors contributing to hypoxic tumor resistance can guide more effective treatment strategies.

#### 3.1.2 Mechanisms of hypoxia-induced resistance

##### 3.1.2.1 Insufficient oxygen

Much of the tumor damage caused by IR is mediated indirectly via the generation of reactive free radicals [73]. These radicals react with oxygen to form ROS, which aggravates radiation damage to DNA, making it irreparable in oxygen-normal tissues [74]. However, under hypoxic conditions, the reaction is limited due to insufficient oxygen, leading to radiation resistance in hypoxic tumor regions (Figure 1). To enhance radiosensitivity in hypoxic tumors, researchers aim to increase oxygen levels using various methods, including hemoglobin transport, erythropoietin stimulation of hemoglobin production [75], or the use of artificial blood substances like perfluorocarbons to deliver oxygen [76,77]. Suppressing oxygen consumption is another strategy to eliminate tumor hypoxia. FDA-approved drugs like Papaverine and Atovaquone alleviate hypoxia by targeting mitochondrial electron transport chain complexes [78,79,80]. Metformin, initially used for type 2 diabetes, also reduces tumor mitochondrial oxygen consumption. Several ongoing clinical trials are exploring metformin as a tumor oxygenating agent (NCT04275713, NCT03510390, NCT02394652, Phase II).

In addition to insufficient oxygen supply, hypoxia-adapted phenotypic modulations are also reasons for tumor cells’ radiotherapy resistance.

##### 3.1.2.2 Hypoxia-inducible factor-1 (HIF-1)

HIF-1 is a crucial transcription factor that regulates cellular responses to hypoxia. It is a heterodimer comprised of one of three hypoxia-inducible alpha subunits (HIF-1α, HIF-2α, or HIF-3α) and a constitutively expressed subunit, HIF-1β [82]. Among these, HIF-1 is the most significant, formed by HIF-1α and HIF-1β. HIF-1α is expressed in nearly all cells, with its expression tightly regulated by oxygen levels. The biological function of HIF-1 is activated only when HIF-1α binds to HIF-1β. Under hypoxic conditions, HIF-1α is stably expressed and translocates to the nucleus, where it regulates downstream gene expression by binding to hypoxia-responsive elements of target genes. Elevated levels of HIF-1α confer radioresistance through various pro-cancer mechanisms, including cell cycle arrest, altered energy metabolism, autophagy, epithelial-mesenchymal transition (EMT), apoptosis, DDR, and modulation of tumor immunity [81]. Studies indicate that HIF-1α influences cell cycle modulation and promotes nonhomologous end joining during post-radiation DNA repair, reducing apoptotic cell death in cervical carcinoma, oral squamous cell carcinoma, and prostate cancer cells [82,83,84].

The serine peptidase inhibitor Kazal type 1 is secreted in a HIF-dependent manner, reducing radiation-induced DNA damage and enhancing radioresistance in adjacent cancer cells via epidermal growth factor receptor and nuclear factor erythroid 2-related factor 2 [85]. HIF-1α also induces the expression of autophagy-related genes such as beclin and LC3-II, activating autophagy [86,87]. Hypoxia-induced autophagy may diminish radiosensitivity by helping cancer cells adapt to metabolic stress from limited oxygen and nutrient availability [88,89,90], particularly in hepatoma, colon cancer, lung cancer, breast cancer, and glioma [86,87,88,91,92]. However, irradiated hypoxic cancer cells can sometimes undergo autophagic death [88,89,93], suggesting that hypoxia-induced autophagy may have contrasting roles in radiotherapy resistance depending on the context.

Research has shown that HIF-1-deficient tumors exhibit greater sensitivity to radiotherapy compared to their wild-type counterparts [94,95,96]. Given its pivotal role in overcoming radiotherapy resistance due to tumor hypoxia, numerous HIF-1 inhibitors are currently under development [97]. Despite extensive clinical trials conducted to date [98,99,100,101], no specific HIF1 inhibitors have been adopted in general clinical practice [74,102]. Therefore, further research is warranted to improve the radiosensitivity of hypoxic tumors through HIF-1 inhibition.

##### 3.1.2.3 Warburg effect

Hypoxic tumor zones adapt to oxygen and nutrient scarcity by shifting energy metabolism from mitochondrial oxidative phosphorylation to glycolysis, known as the Warburg effect. This metabolic switch forces hypoxic tumor cells to generate ATP anaerobically, resulting in decreased ROS production and intracellular accumulation of reduced glutathione (GSH) [103,104]. Increased GSH levels may enhance the radioresistance of hypoxic tumors by boosting their antioxidant capacity [105]. Additionally, this metabolic reprogramming can lead to acidosis, promoting malignant growth and further enhancing radiotherapy resistance [106,107,108]. Researchers propose designing radiosensitizers that activate in low pH environments to target hypoxic tumors specifically. The metabolic switching in hypoxic tumor cells is primarily driven by HIF-1-mediated expression of critical enzymes and regulators of carbohydrate metabolism, including hexokinase 2, pyruvate dehydrogenase kinase 1, and glucose transporter 1, all integral to the Warburg effect [103,104,109]. Other factors, such as the pentose phosphate pathway [110], tumor necrosis factor receptor-associated protein1 [111,112,113], the cellular energy sensor AMP-activated protein kinase [114], and certain microRNAs and circular RNAs [115], also influence cancer cell energy metabolism in response to hypoxia, affecting the radiotherapeutic outcomes for hypoxic tumors.

##### 3.1.2.4 Heat shock transcription factor 1 (HSF1)

HSF1 is responsible for upregulating inducible heat shock proteins (HSP90, HSP70, and HSP27) and is activated in cancer cells under hypoxic stress [116]. These proteins play a protective role, shielding tumor cells from post-radiation cell death and replicative senescence, thereby contributing to radioresistance [116]. HSF1 is crucial for processes such as G2 cell cycle arrest following radiation, the repair of double-stranded DNA breaks [117], the intrinsic promotion of EMT [118], and the upregulation of multidrug resistance 1 expression, which can extrude small molecule radiosensitizers from hypoxic tumor cells [119]. Targeting the mechanisms mediated by HSF1 and HSPs could enhance the sensitivity of hypoxic tumor cells to radiotherapy.

##### 3.1.2.5 CSCs

CSCs exhibit increased resistance to radiotherapy compared to non-stem cancer cells, allowing them to survive treatment and potentially trigger cancer relapse [120]. Hypoxic tumors facilitate EMT through mechanisms such as tumor acidosis and exosome release from hypoxia-stressed cells, which increases the population of radio-resistant CSC-like phenotypes [121]. Central to this process are HIFs, which activate key survival pathways, including transforming growth factor (TGF)-β, Notch, Hedgehog, Wnt/β-catenin, and phosphatidylinositol 3-kinase (PI3K)/AKT/mTOR [122]. These pathways support CSC survival in hypoxic conditions by preserving their phenotype, facilitating self-renewal, and promoting aggressive migration, culminating in heightened radioresistance [123]. Thus, developing radiosensitizing agents targeting hypoxic tumor cells may enhance their sensitivity to radiotherapy and reduce the pool of radioresistant CSCs.

**Figure 3:**
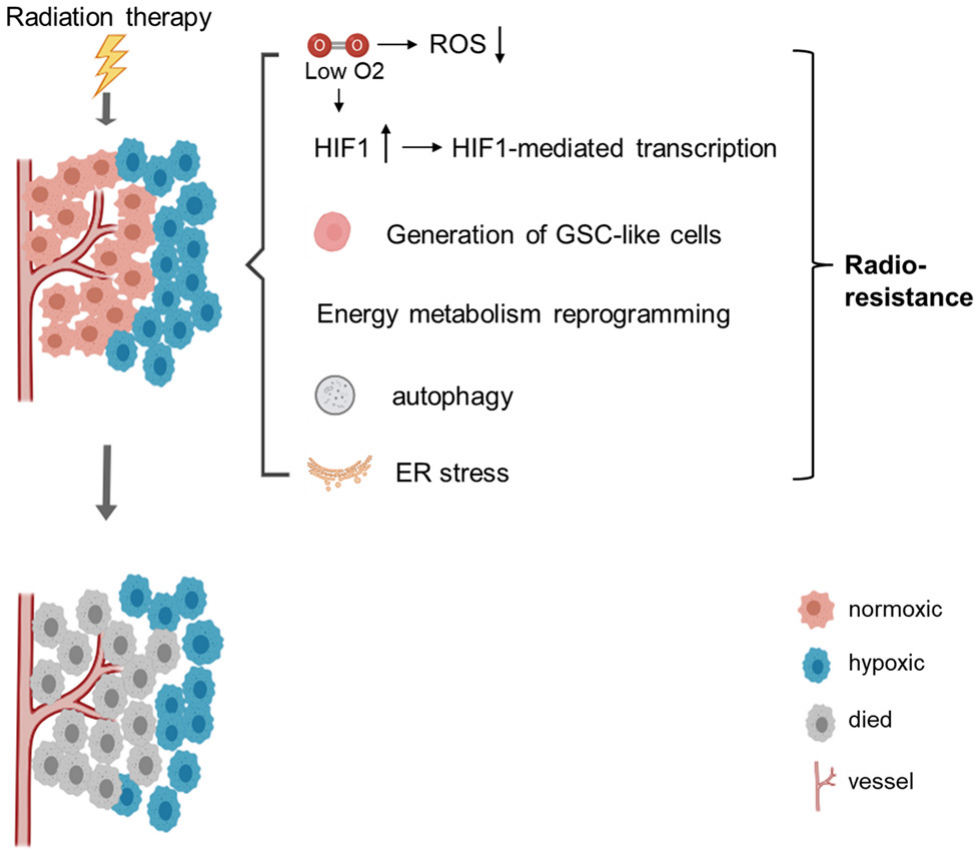
Schematic representation of intratumoral hypoxia and the key hypoxia-induced responses driving the radioresistance of hypoxic tumors. These responses include reduced ROS production due to diminished oxygen availability, upregulation of HIF-1 expression and its oncogenic effects on downstream genes, generation of GSC-like cells, reconfiguration of energy metabolism, autophagy, and ER stress.

Other cellular mechanisms and regulatory pathways in response to hypoxia in cancer cells include hypoxia-induced endoplasmic reticulum (ER) stress and the expression of glucose-regulated proteins [124,125], epigenetic regulation, and exosome generation. These hypoxia-induced responses contribute to the radioresistance of cancer cells. A deeper understanding of the mechanisms behind hypoxic tumor resistance to radiotherapy has revealed several strategies to enhance radiosensitivity. These include artificial oxygenation to improve oxygen delivery to hypoxic tumors [126,127], the use of hypoxia-activated prodrugs that generate cytotoxic agents or radiosensitizers, and the application of hypoxia positron emission tomography (PET) imaging in radiation therapy [128,129]. While these strategies present promising avenues for targeting hypoxic tumors, a single approach is insufficient. Thus, further exploration of the complex cellular interactions and mechanisms within adaptively hypoxic tumor cells is essential to develop synergistic therapeutic strategies (Figure 3).

### 3.2 CSCs

CSCs are a distinct subset of cells found in tumorous tissues that possess the ability to both renew themselves and give rise to a heterogeneous population of tumor cells [130]. These cells display several characteristics, including enhanced DNA damage repair, efficient ROS scavenging, prolonged dormancy, reduced cell adhesion, and potent immunosuppressive properties. Consequently, CSCs play a pivotal role in tumor metastasis, recurrence, radiotherapy failure, and the overall poor prognosis observed in cancer patients [131,132].

**Figure 4:**
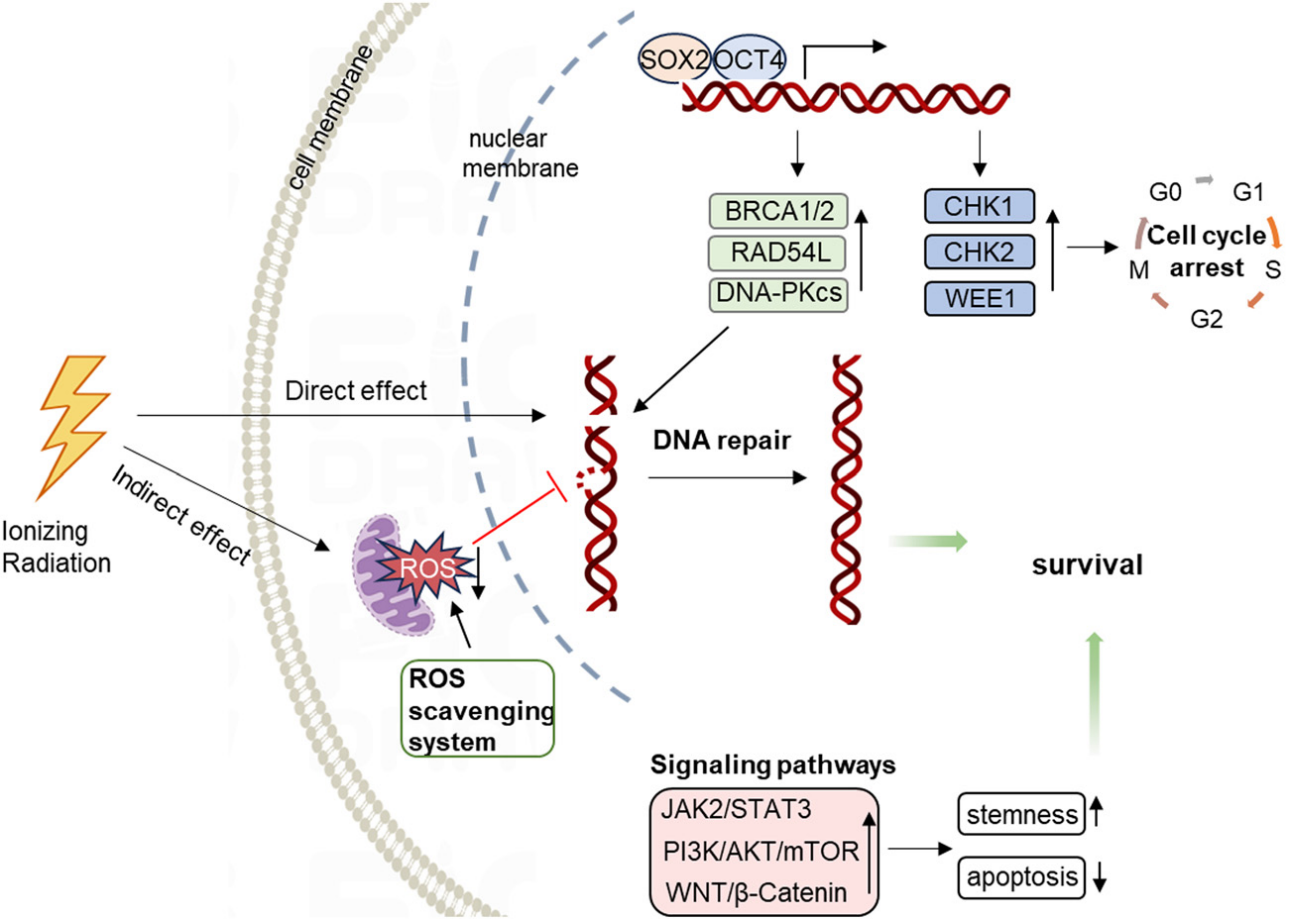
GSCs exhibit radiotherapy resistance due to enhanced DNA repair capabilities, strong ROS scavenging capacity, and multiple signaling pathways maintaining stemness and inhibiting apoptosis.

The radioresistance of CSCs is attributed to their enhanced ability to repair DNA damage and regulate ROS levels (Figure 4). When tumor tissues are exposed to IR, CSCs activate critical checkpoint pathways, such as ATR-CHK1 and ATM-CHK2, upregulating genes involved in DNA repair and cell cycle arrest to facilitate damage repair [35,133,134]. The transcription factor c-Myc serves as a master regulator of various cellular programs, significantly contributing to the radioresistance of stem-like populations in nasopharyngeal carcinoma (NPC) by upregulating DNA damage checkpoints CHK1 and CHK2 [135,136]. Oct4, a CSC marker and transcription factor, confers radioresistance to head and neck squamous cell carcinoma cells by regulating the cell cycle checkpoint kinases CHK1 and WEE1 and HR repair genes PSMC3IP and RAD54L. Combining radiotherapy with PARP inhibitors may induce synthetic lethality in Oct4-deregulated tumors [137]. SOX2, another crucial CSC biomarker, is essential for stem cell self-renewal, reprogramming, and homeostasis [138]. It also contributes to radioresistance by inducing cell cycle arrest, enabling cancer cells to evade DNA damage checkpoints [139]. THOC2 and THOC5 enhance the radioresistance of TNBC by increasing stemness through SOX2 [140]. Musashi1, a CSC marker, regulates DNA-PKcs expression, leading to enhanced DNA repair responses and subsequent radioresistance in GSCs [141]. Ubiquitin-Specific Protease 1, highly expressed in GSCs, stabilizes DDR regulators ID1 and CHK1, thereby promoting radioresistance [142].

Multiple signaling pathways facilitate both stemness and radioresistance in tumor cells. The activation of the JAK2/STAT3 pathway enhances colorectal cancer stemness by upregulating cyclin D2 expression following radiotherapy, minimizing DNA damage accumulation and contributing to radioresistance [143]. The PI3K/AKT/mTOR pathway reduces apoptosis when activated, inducing radioresistance in prostate CSCs [144]. Tribble 2 also activates the mTOR pathway, promoting stemness and radioresistance in esophageal squamous cell carcinoma [145]. The TGF-β and WNT/β-Catenin pathways enhance stemness and increase radioresistance in various tumors, including breast and colon cancers and salivary adenoid cystic carcinoma [146,147,148]. Exosomal miR-19b downregulates FBXW7, activating the WNT/β-Catenin pathway and enhancing both stemness and radioresistance in colorectal cancer CSCs [149]. The Forkhead Box Q1/Sirtuin 1/β-Catenin axis and the Ecotropic Virus Integration Site 1/β-Catenin axis also mediate stemness and radioresistance in colorectal cancer [150,151]. In glioblastoma, the cyclin-like protein Spy1 endows cancer cells with self-renewal capabilities and downregulates CAP-Gly Domain-Containing Linker Protein 3, whose expression promotes glycolytic flux, leading to GSC radioresistance [152]. NRP1, a transmembrane glycoprotein, enhances stemness and potentiates radioresistance in breast cancer cells by reducing radiation-induced apoptosis [153]. Integrin β1 increases stemness in oral squamous carcinoma cells and induces radioresistance by suppressing radiation-induced apoptosis [154].

In addition to their enhanced DNA repair capabilities, CSCs can prevent DNA damage by efficiently clearing ROS. Some CSCs have developed highly effective ROS scavenging systems to maintain low ROS levels, with antioxidant enzymes such as superoxide dismutase, glutathione peroxidase, and catalase exhibiting significant activity [155,156,157]. Elevated ROS levels can activate HIF, triggering pro-survival and developmental pathways like Notch, WNT, and Hedgehog, which support CSC survival [158]. Research has also identified a negative feedback loop between ROS and COX-2 in CSCs, where increased ROS induces COX-2 expression, which in turn reduces ROS accumulation, promoting CSC enrichment and metastasis [159]. Autophagy, crucial for maintaining cellular homeostasis, is recognized as a key resistance mechanism in metastatic prostate CSCs, significantly enhancing ROS clearance [160,161]. Consequently, CSCs exhibit heightened sensitivity to fluctuations in the oxidant/antioxidant balance, acquiring resistance under both low and elevated ROS conditions.

Furthermore, CSCs primarily remain in a quiescent state, residing in the highly resistant G0 phase during the cell cycle, which minimizes radiation-induced DNA damage [162]. IR can induce a radioresistant stemness phenotype in glioma stem cells in GBMs by promoting autophagy through the Wnt/β-catenin pathway [163]. Similarly, autophagy protects leukemia stem cells from radiotherapy toxicity, enhancing their survival [164]. Studies indicate that inhibiting autophagy-related proteins, specifically SLC7A5/LAT1 and ATG5, increases radiosensitivity in head and neck squamous cell carcinomas (HNSCC) [165]. Solid tumor CSCs predominantly reside in hypoxic niches [166], which may shield them from radiation damage through reduced ROS production, decreased DNA damage, and the activation of the HIF signaling pathway [167,168]. Additionally, CSCs can emit immunosuppressive signals, modifying their microenvironment into an immunosuppressive milieu that fosters tumor growth and contributes to radioresistance [169].

Recent research underscores the pivotal role of CSCs in tumor radioresistance. By enhancing our understanding of potential therapeutic targets for CSC radiosensitization, we can develop more effective and safer combination strategies to improve cancer patients’ life expectancy and treatment outcomes.

Clarifying the regulatory mechanisms and identifying CSC biomarkers is a primary focus of current research. In preclinical studies, significant efforts have been made to restore radiosensitivity by targeting CSCs. For example, DNA-PK stabilizes SOX2, maintaining GSC stemness. The DNA-PK inhibitor NU7441 effectively reduces stem cell sphere formation and sensitizes tumors to radiotherapy *in vivo* [28]. Additionally, delivering miR-145, which targets multiple stemness-related transcription factors, reduces stemness and reverses the radioresistance of colorectal CSCs [170]. Combining radiotherapy with glimepiride, a type 2 diabetes treatment, disrupts GSC maintenance and sensitizes tumors to radiation by reducing glycolysis [152]. BEZ235, a dual PI3K/mTOR inhibitor, effectively sensitizes prostate CSCs to radiotherapy by decreasing stemness [144]. Moreover, methyltransferase-like 14 and miR-99a-5p downregulate Tribble 2, while Tribble 2-induced activation of the mTOR pathway can be inhibited by histone deacetylase 2 (HDAC2) inhibitors, restoring radiosensitivity in esophageal squamous CSCs [145]. Despite these preclinical efforts, few clinical trials have investigated the combination of radiotherapy with CSC-targeting therapies. One example is a phase I study (NCT01068327) that evaluated the safety and efficacy of nelfinavir, an Akt inhibitor, combined with SBRT for locally advanced, borderline, or unresectable pancreatic adenocarcinoma. The trial concluded that concurrent SBRT plus nelfinavir was tolerable and safe for patients with locally advanced pancreatic cancer, although the efficacy of this combination requires further investigation [171].

Despite numerous challenges, researchers remain committed to innovative approaches to eradicate CSCs and enhance their responsiveness to radiotherapy. Future studies on CSC characteristics – including novel markers, signaling pathways, and the TME – are anticipated to be a major focus. Although high-quality clinical trials confirming the efficacy of these strategies are currently lacking, ongoing research provides optimism for developing more effective methods to eliminate CSCs and overcome radiotherapy resistance.

### 3.3 Immune microenvironment

The immune system is the primary defense against cancer and plays a critical role in cancer progression [66]. In recent years, immunotherapy has emerged as a leading strategy in cancer treatment, yet its success is often limited to specific patient subsets, particularly in non-small cell lung cancer (NSCLC). Radiotherapy can enhance the immune response to tumors through multiple mechanisms, including modifying immunosurveillance by altering neoantigen expression, inducing the abscopal effect [172,173], activating the cGAS-STING pathway, and increasing type I interferon transcription, thereby boosting the innate immune response [174]. Consequently, the combination of radiotherapy and immunotherapy is gaining recognition as a promising therapeutic approach. However, it is essential to recognize that radiotherapy can simultaneously promote anti-tumor immune responses and activate immunosuppressive mechanisms, potentially leading to therapy resistance.

When tumor cells cannot repair radiation-induced DNA damage, they enter a state of senescence [175]. In this state, they release immunosuppressive cytokines, such as TGF-β1 and chemokine (C-C motif) ligand 2, which attract myeloid cells with immunosuppressive phenotypes, including myeloid-derived suppressor cells and M2-like tumor-associated macrophages. These cells inhibit CD8+ T cell activation and function, facilitating tumor immune resistance and diminishing radiotherapy efficacy [176,177,178]. The excessive activation of the phosphatidylinositol 3-kinase (PI3K)/AKT pathway post-radiotherapy is closely associated with tumor radioresistance. Key downstream effectors of AKT, including NF-κB and mTOR, enhance cell survival by strengthening the DDR and regulating autophagy and apoptosis [179,180]. Furthermore, the PI3K-AKT pathway is instrumental in sustaining HIF-1α transcription in tumors [181]. Targeting the PI3K-AKT-mTOR pathway could reduce tumor hypoxia and induce G2/M phase arrest in cells sensitive to radiation-induced DNA damage [182,183].

IR also increases programmed death ligand 1 (PD-L1) expression through various mechanisms, undermining the cytotoxic effects of CD8+ CTLs [184,185]. Combining radiation with anti-PD-L1 therapy may curb immune evasion, enhancing the anti-cancer efficacy of radiotherapy [186]. PD-L1 interacts with programmed death-1 (PD-1), leading to T cell apoptosis, dysfunction, and exhaustion, thereby inhibiting the activation and proliferation of tumor antigen-specific CD8+ T cells, which facilitates tumor immune escape [187]. Pembrolizumab, a PD-1 inhibitor, blocks this interaction [188]. Recently, Pembrolizumab has advanced significantly as an immunotherapy for various tumors, and its combination with radiotherapy as a radiosensitizer is receiving increasing attention [189]. While radiotherapy can eliminate local tumor cells, Pembrolizumab may enhance the abscopal effect by activating systemic immune responses. Clinical studies are currently investigating the combination of Pembrolizumab and radiotherapy for multiple solid tumors, including NSCLC, melanoma, and HNSCC (Table 1) [190,191,192]. Moreover, combining radiotherapy with anti-CTLA-4 and related immunomodulatory agents may yield synergistic benefits [193,194].

**Table 1:** Registered ongoing clinical trials of small-molecule chemical radiosensitizers in the past 5 years

Trial ID	Intervention	Conditions	Phase	Study start
NCT04381806	5-ALA	Solid tumor	I	2020-07-30
NCT04634877	Cisplatin	High-risk endometrial cancer	III	2021-01-10
NCT05626829	Tranilast	NPC	II	2022-07-20
NCT06103617	Penicillamine	Recurrent head and neck cancer	II	2023-11-15
NCT03946202	Hydrogen peroxide	Advanced/recurrent breast cancer	II	2020-06-16
NCT06142318	Pirfenidone	HNSCC	II	2023-11-15
NCT04683679	Pembrolizumab	Triple-negative or hormone-receptor positive/​Her2 negative breast cancer	II	2021-04-21

Recent findings reveal that elevated expression of the tumor-specific E3 ligase TRIM7 correlates with poor outcomes in NPC due to its role in impairing mitochondrial DNA release, disrupting STING/STING-dependent interferon signaling. This disruption compromises CD8+ T cell-mediated anti-tumor immune responses and contributes to radiation therapy resistance [195]. Understanding the interplay between the positive and negative effects of radiotherapy within the tumor immune environment is crucial for developing strategic combinations of radiotherapy and immunotherapy to enhance overall treatment efficacy.

### 3.4 Cancer‑associated fibroblasts (CAFs)

CAFs are a critical and adaptable cell population in the TME. Through dynamic interactions with tumor cells, they provide structural support and functional assistance, promoting tumor progression and therapy resistance [196]. CAFs induce EMT and enhance the CSC phenotype by releasing paracrine exosomes that activate TGF-β signaling [197,198,199], thereby increasing tumor radiation resistance. CAF-derived exosomes contain elevated levels of miR-935p compared to those from normal fibroblasts, leading to decreased radiation-induced apoptosis in colorectal cancer cells [200]. In esophageal squamous cell carcinoma, chemokine CXCL1 secreted by CAFs confers radioresistance by regulating the DDR in a ROS-dependent manner [201]. Similarly, in NPC, CAFs enhance radioresistance and reduce DNA damage through IL-8 secretion, activating NF-κB signaling [202]. Furthermore, exosomes secreted by CAFs interact with tumor cells via retinoic acid-inducible gene-I (RIG-I), amplifying radioresistance [203]. CAFs also diminish the efficacy of radiotherapy by fostering an immunosuppressive environment enriched with immunosuppressive cells and inhibiting effector immune cells [204,205]. A transcriptomic analysis of CAFs following radiotherapy in an NSCLC model revealed the upregulation of nine distinct MDM2 transcripts. Increased expression of these MDM2 variants correlates with lung radiosensitivity [206], indicating a potential role for CAFs in mediating radioresistance post-therapy. Although CAFs significantly influence the TME and contribute to radioresistance, the nuances of how radiotherapy affects CAFs and their subsequent interactions with the TME remain underexplored. An in-depth study of this interplay is essential, as it holds potential for guiding innovative therapeutic approaches beyond merely inhibiting or eradicating tumor cells.

The complexity of the TME enables cancer cells to develop resistance to radiotherapy through multiple mechanisms. To overcome this resistance, researchers are exploring ways to target these microenvironmental components. Currently, several small molecule drugs are undergoing preclinical studies (Table 1). Tranilast, originally used as an anti-allergic drug, has recently been found to possess anti-tumor properties, particularly when combined with radiotherapy, where it exhibits radiosensitizing effects [207]. Its potential as a radiosensitizer is being actively explored, particularly regarding its role in modulating the TME and combating fibrosis. Tranilast inhibits TGF-β activity, a key factor in the TME that promotes tumor cell proliferation, invasion, and metastasis, and plays a critical role in radioresistance [208]. By blocking the TGF-β signaling pathway, tranilast reduces tumor cell radioresistance. It also inhibits CAF activity, diminishing the supportive environment for tumor cells and enhancing the effectiveness of radiotherapy. Although clinical studies on tranilast as a radiosensitizer are still in early stages, preliminary results are promising. For instance, in locally advanced tumors such as head and neck cancer and NSCLC, early findings suggest that tranilast can improve local tumor radiosensitivity and enhance overall patient survival [202,209].

However, radiotherapy itself can significantly affect the TME by inducing immune cell infiltration, promoting fibrosis, and activating TGF-β signaling. These changes may enhance the short-term efficacy of radiotherapy but could also contribute to long-term tumor recurrence and resistance. To effectively overcome tumor radioresistance, future research must delve deeper into the factors within the TME that contribute to radiotherapy resistance and their interactions. Identifying these factors will be crucial for discovering new targets to enhance the efficacy of radiotherapy.

## 4 Other factors

### 4.1 Epigenetic factors

**Figure 5:**
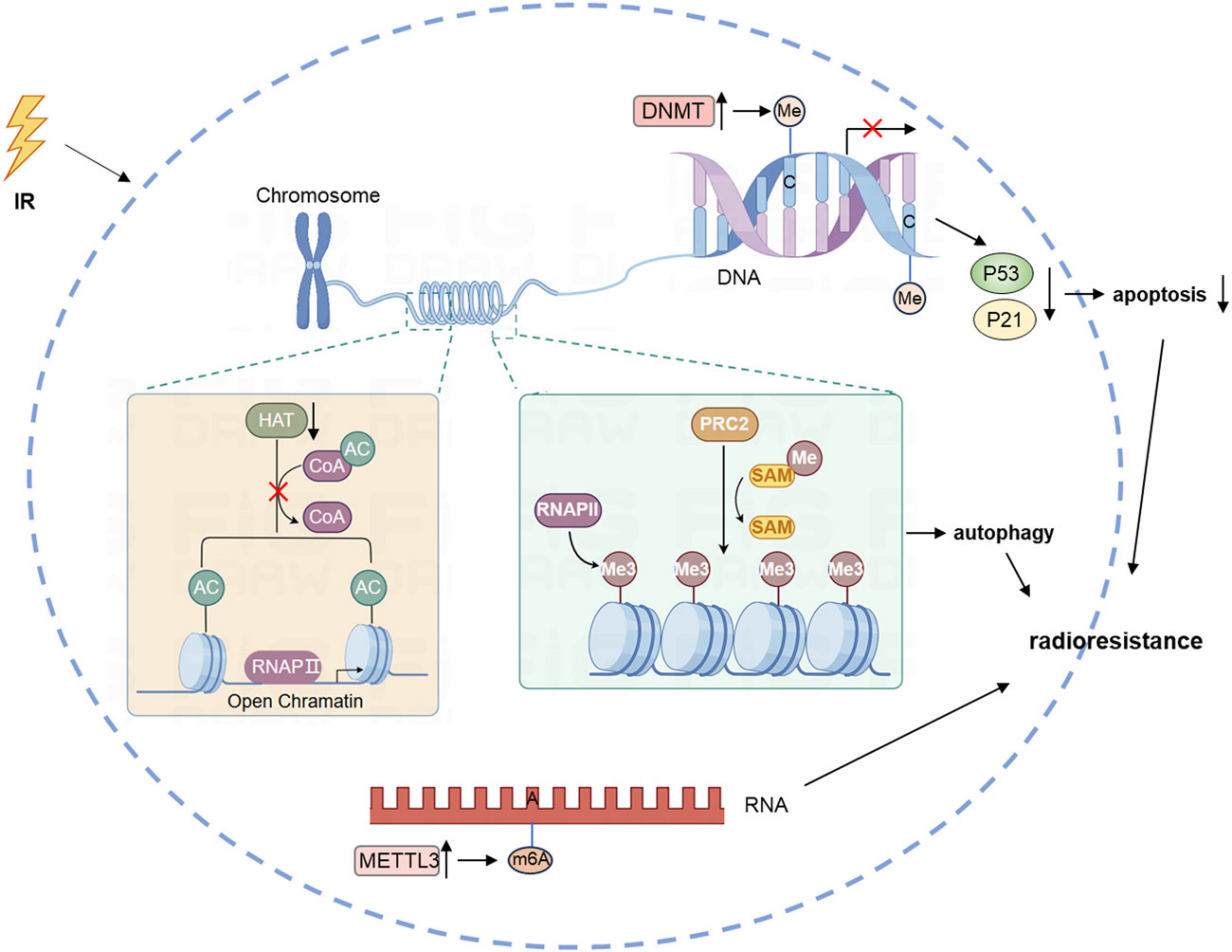
IR can affect cellular epigenetics by acting on DNA, leading to increased methylation levels that impact the transcription of genes like p53. IR also influences histone methylation, acetylation, and RNA adenosine methylation levels in tumor cells by acting on specific enzymes. These radiation-induced responses ultimately contribute to radioresistance.

Epigenetics encompasses mechanisms that influence gene expression without altering the DNA sequence. These mechanisms include DNA methylation, histone modifications (such as acetylation and methylation), and the regulation of non-coding RNAs [210] (Figure 5). Numerous studies have demonstrated a strong correlation between epigenetic modifications and the resistance of tumor cells to radiotherapy [211].

#### 4.1.1 DNA methylation

DNA methylation involves adding a methyl group to the fifth carbon of cytosine within CpG dinucleotides, a process facilitated by DNA methyltransferases (DNMTs). Radiotherapy can induce changes in DNA methylation or demethylation and affect DNMT activity [212]. For example, in a rat breast cancer study, radiation-induced hypermethylation, mediated by the polycomb repressive complex 2 (PRC2), led to cancer cell dedifferentiation and contributed to radiation-induced breast cancer [213]. Hypermethylation of specific gene promoter regions can silence genes and enhance cancer cell resistance to radiation. Silencing DNA methyltransferase 3B (DNMT3B) has been shown to restore p53 and p21 function through demethylation, resulting in cell cycle arrest and apoptosis [214]. IR can increase DNMT3B levels and methylate p53 and p21, promoting radiation resistance in NPC [215].

#### 4.1.2 Histone modification

Histone modifications, including methylation, acetylation, phosphorylation, and ubiquitination, are crucial for regulating gene expression and are closely tied to transcription and DNA repair [216,217,218]. Radiation significantly affects histone modifications in tumor cells [219]. A notable modification is the trimethylation of histone H3 at lysine 27 (H3K27me3), associated with chromatin condensation and impacting DNA DSB repair. In diffuse intrinsic pontine glioma (DIPG), H3K9me3 levels increase after radiotherapy. A strategy combining radiotherapy with a G9a inhibitor aims to reduce H3K9me3 and DSB repair [220]. While radiation eliminates many cancer cells, it can also induce radioresistance and more aggressive epigenetic phenotypes. Upregulation of the chemokine CXCL12, mediated by histone modification in its promoter, may drive the development of a resistant phenotype during treatment [221]. Research has also linked radiation-induced autophagy to histone methylation, identifying H4K20me3 as critical in inducing autophagy post-irradiation. This autophagy acts as a protective mechanism for NSCLC cells, and inhibiting autophagy-associated histone modifications can enhance cell death following radiation. The broad-spectrum methyltransferase inhibitor 3-deazaneplanocin A (DZNep) significantly inhibits H4K20me3, enhancing radiosensitivity [222].

Histone acetylation activates gene transcription by acetylating lysine residues on histone tails. Aberrant histone acetylation has been linked to radiation resistance [201]. One study found that radiation-resistant populations exhibited widespread histone deacetylation and alterations in the activity of HDACs and histone acetyltransferases. Given the variability of HDAC activity among individuals, assessing tumor HDAC activity prior to radiotherapy is crucial. Patients with high HDAC activity may benefit from radiosensitization with HDAC inhibitors (HDACi) [223].

#### 4.1.3 RNA methylation

Post-transcriptional modifications of RNA molecules are prevalent, with over a hundred known modifications, including *N*6-methyladenine (m6A), 5-methylcytosine, *N*1-methyladenosine, and M7G, with methylation being the most common. YTH domain containing 2 (YTHDC2) is an m6A-binding protein [224]. In NPC, YTHDC2 is overexpressed in radioresistant cells. Knockout of YTHDC2 enhances the therapeutic effects of radiotherapy both *in vitro* and *in vivo*, whereas its overexpression in radiation-sensitive NPC cells has the opposite effect [225]. In hypopharyngeal squamous cell carcinoma, methyltransferase-like 3 mediates m6A methylation and stabilizes the expression of circCUX1, a specific circRNA. Knocking down circCUX1 increases hypopharyngeal cancer cells’ sensitivity to radiotherapy. Furthermore, circCUX1 inhibits caspase-1 expression, reducing the release of inflammatory cytokines and increasing tolerance to radiotherapy [226].

Chromatin structure has also emerged as a potential target in radiation therapy. Chromatin remodeling complexes, such as the SWI/SNF complex, modulate DNA accessibility, affecting gene expression and potentially contributing to radioresistance [202,203,204]. Additionally, non-coding RNAs, particularly microRNAs (miRNAs) and long non-coding RNAs (lncRNAs), have been significantly associated with radiation resistance [205,206]. The interplay between epigenetic alterations and radioresistance likely varies across cancer types, emphasizing the need to understand these processes for effective radiosensitizing strategies.

The study of epigenetics is crucial in cancer research. Advances have revealed that certain epigenetic modifiers – such as HDAC inhibitors, DNMT inhibitors, EZH2 inhibitors, and BET inhibitors – can act as radiation sensitizers [227]. These agents impair DNA damage repair, disrupt the cell cycle, and increase oxidative stress, thereby enhancing the anti-tumor efficacy of radiotherapy.

### 4.2 Non-B-DNA

DNA, the genetic material of human cells, typically exists as double-stranded B-DNA, characterized by Watson–Crick base pairing. However, under certain conditions, repetitive DNA motifs can adopt non-B DNA forms, such as self-annealed hairpins, Z-DNA, three-stranded triplexes (H-DNA), or four-stranded guanine quadruplex structures (G4 DNA) [228,229]. Among these, G4 DNA is the most extensively studied non-B DNA structure.

G4 DNA plays a crucial role in carcinogenesis and the malignant phenotypes of cancer cells [230]. Research indicates that G4 DNA can fold into a quadruplex structure that is less sensitive to IR compared to B-DNA [231]. During radiotherapy, the planar G-quadruplex of G4 DNA can shield free radicals induced by IR, protecting genomic regions rich in G4 DNA from radiation-induced breaks. This process can be further regulated by G4 stabilizers, helping the human genome resist radiation-induced damage [232]. In cancer tissues, the proportion of G4 DNA is upregulated, accelerating genomic instability [233]. The presence of G4 DNA is associated not only with cancer development and tumor malignancy but also with the effectiveness of radiotherapy.

Controlling non-B DNA structures may offer a novel strategy for enhancing radiotherapy sensitivity. One extensively studied approach involves developing small-molecule drugs that specifically recognize and stabilize these structures. For instance, G-quadruplex stabilizers, such as TMPyP4, have been investigated for their potential in radiosensitization [234,235]. Another strategy targets proteins that bind to non-B DNA structures; proteins like Topoisomerase and RPA can stabilize these structures, and targeting their functions could indirectly influence non-B DNA formation [236,237]. Despite numerous laboratory studies suggesting that non-B DNA structures can enhance radiotherapy sensitivity, most research remains limited to cell and animal models. The translation of these findings into clinical applications and the development of safe, effective drugs require further exploration and validation.

## 5 Discussion

Radiotherapy plays a major role in cancer treatment, yet radioresistance poses significant challenges to achieving optimal therapeutic outcomes. This review has examined the various factors and pathways contributing to tumor radioresistance. Mechanisms such as DNA repair and cell cycle dynamics are critical biological factors driving resistance. Moreover, the TME – characterized by hypoxia, immune evasion, and CSCs – complicates treatment efficacy. Molecular mechanisms, including aberrant signaling pathways, epigenetic modifications, and non-B-DNA, also significantly contribute to radioresistance.

To address tumor resistance to radiotherapy, combining it with targeted drugs that inhibit DNA repair, modulate the TME, or disrupt signaling pathways may overcome this challenge. For instance, PARP inhibitors have been shown to enhance radiotherapy effects by blocking DNA repair mechanisms. Additionally, optimizing the dose distribution and timing of radiotherapy can maximize tumor cell destruction while minimizing damage to healthy tissues.

Recent advancements in immunotherapy have demonstrated immense potential, particularly in cancers where traditional therapies are less effective. Consequently, combining immunotherapy with radiotherapy is considered one of the most promising strategies. Radiotherapy can enhance the exposure of tumor antigens, facilitating immune recognition and attack, while immune checkpoint inhibitors can counteract the immunosuppressive effects of the TME. This combined approach offers potential synergistic effects by activating the immune system while simultaneously destroying tumor cells. An in-depth understanding of the interplay between the advantages and disadvantages of radiotherapy within the tumor immune milieu could pave the way for strategic combinations of radiotherapy and immunotherapy, ultimately enhancing the overall efficacy of treatment.

Since radioresistance arises from multiple factors, different tumor types may develop resistance through distinct regulatory pathways. Overcoming this resistance necessitates tailored molecular interventions or combination therapies specific to each tumor subtype. A deeper understanding of the molecular mechanisms underlying radioresistance, along with insights into their interactions with the TME, could improve radiotherapy efficacy in resistant cancers. Furthermore, advancements in genetic marker identification, molecular profiling, enhanced molecular imaging, and functional assays are opening new avenues for predicting treatment responses, driving the field toward more precise and individualized clinical care.

## References

[1] Sung H, Ferlay J, Siegel RL, Laversanne M, Soerjomataram I, Jemal A, et al. Global cancer statistics 2020: GLOBOCAN estimates of incidence and mortality worldwide for 36 cancers in 185 countries. CA Cancer J Clin. 2021;71(3):209–49. 10.3322/caac.21660 33538338

[2] Siegel RL, Miller KD, Wagle NS, Jemal A. Cancer statistics, 2023. CA Cancer J Clin. 2023;73(1):17–48. 10.3322/caac.21763 36633525

[3] Krzyszczyk P, Acevedo A, Davidoff EJ, Timmins LM, Marrero-Berrios I, Patel M, et al. The growing role of precision and personalized medicine for cancer treatment. Technology (Singap World Sci). 2018;6(3–4):79–100. 10.1142/S2339547818300020 PMC6352312 30713991

[4] Kulakowski A. The contribution of Marie Sklodowska-Curie to the development of modern oncology. Anal Bioanal Chem. 2011;400(6):1583–6. 10.1007/s00216-011-4712-1 PMC3093546 21331492

[5] Martin OA, Martin RF. Cancer radiotherapy: Understanding the price of tumor eradication. Front Cell Dev Biol. 2020;8:261. 10.3389/fcell.2020.00261 PMC7193305 32391355

[6] Chandra RA, Keane FK, Voncken FEM, Thomas Jr CR. Contemporary radiotherapy. Lancet. 2021;398(10295):171–84. 10.1016/S0140-6736(21)00233-6 34166607

[7] Balmukhanov SB, Yefimov ML, Kleinbock TS. Acquired radioresistance of tumour cells. Nature. 1967;216(5116):709–11. 10.1038/216709a0 6082487

[8] Kabakov AE, Yakimova AO. Hypoxia-Induced cancer cell responses driving radioresistance of hypoxic tumors: Approaches to targeting and radiosensitizing. Cancers (Basel). 2021;13(5):1102. 10.3390/cancers13051102 PMC7961562 33806538

[9] Azzam EI, Jay-Gerin JP, Pain D. Ionizing radiation-induced metabolic oxidative stress and prolonged cell injury. Cancer Lett. 2012;327(1–2):48–60. 10.1016/j.canlet.2011.12.012 PMC3980444 22182453

[10] Li J, Sun H, Huang Y, Wang Y, Liu Y, Chen X. Pathways and assays for DNA double-strand break repair by homologous recombination. Acta Biochim Biophys Sin (Shanghai). 2019;51(9):879–89. 10.1093/abbs/gmz076 31294447

[11] Gartner A, Engebrecht J. DNA repair, recombination, and damage signaling. Genetics. 2022;220(2):iyab178. 10.1093/genetics/iyab178 PMC9097270 35137093

[12] Baxter JS, Zatreanu D, Pettitt SJ, Lord CJ. Resistance to DNA repair inhibitors in cancer. Mol Oncol. 2022;16(21):3811–27. 10.1002/1878-0261.13224 PMC9627783 35567571

[13] Xiong H, Yan T, Zhang W, Shi F, Jiang X, Wang X, et al. miR-613 inhibits cell migration and invasion by downregulating Daam1 in triple-negative breast cancer. Cell Signal. 2018;44:33–42. 10.1016/j.cellsig.2018.01.013 29339084

[14] Li T, Chen ZJ. The cGAS-cGAMP-STING pathway connects DNA damage to inflammation, senescence, and cancer. J Exp Med. 2018;215(5):1287–99. 10.1084/jem.20180139 PMC5940270 29622565

[15] Maremonti E, Eide DM, Rossbach LM, Lind OC, Salbu B, Brede DA. In vivo assessment of reactive oxygen species production and oxidative stress effects induced by chronic exposure to gamma radiation in Caenorhabditis elegans. Free Radic Biol Med. 2020;152:583–96. 10.1016/j.freeradbiomed.2019.11.037 31805397

[16] Collins PL, Purman C, Porter SI, Nganga V, Saini A, Hayer KE, et al. DNA double-strand breaks induce H2Ax phosphorylation domains in a contact-dependent manner. Nat Commun. 2020;11(1):3158. 10.1038/s41467-020-16926-x PMC7308414 32572033

[17] Blackford AN, Jackson SP. ATM, ATR, and DNA-PK: The Trinity at the heart of the DNA damage response. Mol Cell. 2017;66(6):801–17. 10.1016/j.molcel.2017.05.015 28622525

[18] Khanna KK, Jackson SP. DNA double-strand breaks: signaling, repair and the cancer connection. Nat Genet. 2001;27(3):247–54. 10.1038/85798 11242102

[19] Lord CJ, Ashworth A. The DNA damage response and cancer therapy. Nature. 2012;481(7381):287–94. 10.1038/nature10760 22258607

[20] Carney JP, Maser RS, Olivares H, Davis EM, Le Beau M, Yates 3rd JR, et al. The hMre11/hRad50 protein complex and Nijmegen breakage syndrome: linkage of double-strand break repair to the cellular DNA damage response. Cell. 1998;93(3):477–86. 10.1016/s0092-8674(00)81175-7 9590181

[21] Ho V, Chung L, Singh A, Lea V, Abubakar A, Lim SH, et al. Overexpression of the MRE11-RAD50-NBS1 (MRN) complex in rectal cancer correlates with poor response to neoadjuvant radiotherapy and prognosis. BMC Cancer. 2018;18(1):869. 10.1186/s12885-018-4776-9 PMC6122630 30176843

[22] Shibata A, Jeggo PA. Roles for 53BP1 in the repair of radiation-induced DNA double strand breaks. DNA Repair (Amst). 2020;93:102915. 10.1016/j.dnarep.2020.102915 33087281

[23] Wang S, Wang Y, Liu X, Yang Y, Wu S, Liu Y. SFN enhanced the radiosensitivity of cervical cancer cells via activating LATS2 and blocking Rad51/MDC1 recruitment to DNA damage site. Cancers (Basel). 2022;14(8):1872. 10.3390/cancers14081872 PMC9026704 35454780

[24] Baptistella AR, Landemberger MC, Dias MVS, Giudice FS, Rodrigues BR, da Silva P, et al. Rab5C enhances resistance to ionizing radiation in rectal cancer. J Mol Med (Berl). 2019;97(6):855–69. 10.1007/s00109-019-01760-6 30968159

[25] Watanabe G, Lieber MR, Williams DR. Structural analysis of the basal state of the Artemis:DNA-PKcs complex. Nucleic Acids Res. 2022;50(13):7697–720. 10.1093/nar/gkac564 PMC9303282 35801871

[26] Dibitetto D, Marshall S, Sanchi A, Liptay M, Badar J, Lopes M, et al. DNA-PKcs promotes fork reversal and chemoresistance. Mol Cell. 2022;82(20):3932–42 e6. 10.1016/j.molcel.2022.08.028 PMC9588680 36130596

[27] Chen Y, Jiang T, Zhang H, Gou X, Han C, Wang J, et al. LRRC31 inhibits DNA repair and sensitizes breast cancer brain metastasis to radiation therapy. Nat Cell Biol. 2020;22(10):1276–85. 10.1038/s41556-020-00586-6 PMC7962994 33005030

[28] Fang X, Huang Z, Zhai K, Huang Q, Tao W, Kim L, et al. Inhibiting DNA-PK induces glioma stem cell differentiation and sensitizes glioblastoma to radiation in mice. Sci Transl Med. 2021;13(600):eabc7275. 10.1126/scitranslmed.abc7275 34193614

[29] Guo Z, Wang YH, Xu H, Yuan CS, Zhou HH, Huang WH, et al. LncRNA linc00312 suppresses radiotherapy resistance by targeting DNA-PKcs and impairing DNA damage repair in nasopharyngeal carcinoma. Cell Death Dis. 2021;12(1):69. 10.1038/s41419-020-03302-2 PMC7801696 33431817

[30] Wang YH, Guo Z, An L, Zhou Y, Xu H, Xiong J, et al. LINC-PINT impedes DNA repair and enhances radiotherapeutic response by targeting DNA-PKcs in nasopharyngeal cancer. Cell Death Dis. 2021;12(5):454. 10.1038/s41419-021-03728-2 PMC8105365 33963177

[31] Sudhanva MS, Hariharasudhan G, Jun S, Seo G, Kamalakannan R, Kim HH, et al. MicroRNA-145 impairs classical non-homologous end-joining in response to ionizing radiation-induced DNA double-strand breaks via targeting DNA-PKcs. Cells. 2022;11(9):1509. 10.3390/cells11091509 PMC9102532 35563814

[32] Lee JH, Paull TT. Activation and regulation of ATM kinase activity in response to DNA double-strand breaks. Oncogene. 2007;26(56):7741–8. 10.1038/sj.onc.1210872 18066086

[33] Ma M, Rodriguez A, Sugimoto K. Activation of ATR-related protein kinase upon DNA damage recognition. Curr Genet. 2020;66(2):327–33. 10.1007/s00294-019-01039-w PMC7073305 31624858

[34] Carruthers R, Ahmed SU, Strathdee K, Gomez-Roman N, Amoah-Buahin E, Watts C, et al. Abrogation of radioresistance in glioblastoma stem-like cells by inhibition of ATM kinase. Mol Oncol. 2015;9(1):192–203. 10.1016/j.molonc.2014.08.003 PMC5528679 25205037

[35] Zhang P, Wei Y, Wang L, Debeb BG, Yuan Y, Zhang J, et al. ATM-mediated stabilization of ZEB1 promotes DNA damage response and radioresistance through CHK1. Nat Cell Biol. 2014;16(9):864–75. 10.1038/ncb3013 PMC4150825 25086746

[36] Desai A, Webb B, Gerson SL. CD133+ cells contribute to radioresistance via altered regulation of DNA repair genes in human lung cancer cells. Radiother Oncol. 2014;110(3):538–45. 10.1016/j.radonc.2013.10.040 PMC4004669 24440048

[37] Smith J, Tho LM, Xu N, Gillespie DA. The ATM-CHK2 and ATR-CHK1 pathways in DNA damage signaling and cancer. Adv Cancer Res. 2010;108:73–112. 10.1016/B978-0-12-380888-2.00003-0 21034966

[38] Weber AM, Ryan AJ. ATM and ATR as therapeutic targets in cancer. Pharmacol Ther. 2015;149:124–38. 10.1016/j.pharmthera.2014.12.001 25512053

[39] Jin MH, Oh DY. ATM in DNA repair in cancer. Pharmacol Ther. 2019;203:107391. 10.1016/j.pharmthera.2019.07.002 31299316

[40] Hirao A, Cheung A, Duncan G, Girard PM, Elia AJ, Wakeham A, et al. CHK2 is a tumor suppressor that regulates apoptosis in both an ataxia telangiectasia mutated (ATM)-dependent and an ATM-independent manner. Mol Cell Biol. 2002;22(18):6521–32. 10.1128/MCB.22.18.6521-6532.2002 PMC135625 12192050

[41] Ame JC, Spenlehauer C, de Murcia G. The PARP superfamily. Bioessays. 2004;26(8):882–93. 10.1002/bies.20085 15273990

[42] Lord CJ, Ashworth A. PARP inhibitors: Synthetic lethality in the clinic. Science. 2017;355(6330):1152–8. 10.1126/science.aam7344 PMC6175050 28302823

[43] Caldecott KW, Aoufouchi S, Johnson P, Shall S. XRCC1 polypeptide interacts with DNA polymerase beta and possibly poly(ADP-ribose) polymerase, and DNA ligase III is a novel molecular ‘nick-sensor’ in vitro. Nucleic Acids Res. 1996;24(22):4387–94. 10.1093/nar/24.22.4387 PMC146288 8948628

[44] Kim DS, Camacho CV, Nagari A, Malladi VS, Challa S, Kraus WL. Activation of PARP-1 by snoRNAs controls ribosome biogenesis and cell growth via the RNA helicase DDX21. Mol Cell. 2019;75(6):1270–85 e14. 10.1016/j.molcel.2019.06.020 PMC6754283 31351877

[45] Helleday T, Petermann E, Lundin C, Hodgson B, Sharma RA. DNA repair pathways as targets for cancer therapy. Nat Rev Cancer. 2008;8(3):193–204. 10.1038/nrc2342 18256616

[46] Noordermeer SM, van Attikum H. PARP inhibitor resistance: A tug-of-war in BRCA-mutated cells. Trends Cell Biol. 2019;29(10):820–34. 10.1016/j.tcb.2019.07.008 31421928

[47] Donoho G, Brenneman MA, Cui TX, Donoviel D, Vogel H, Goodwin EH, et al. Deletion of BRCA2 exon 27 causes hypersensitivity to DNA crosslinks, chromosomal instability, and reduced life span in mice. Genes Chromosomes Cancer. 2003;36(4):317–31. 10.1002/gcc.10148 12619154

[48] Moynahan ME, Chiu JW, Koller BH, Jasin M. BRCA1 controls homology-directed DNA repair. Mol Cell. 1999;4(4):511–8. 10.1016/s1097-2765(00)80202-6 10549283

[49] Huang A, Garraway LA, Ashworth A, Weber B. Synthetic lethality as an engine for cancer drug target discovery. Nat Rev Drug Discov. 2020;19(1):23–38. 10.1038/s41573-019-0046-z 31712683

[50] Lee JM, Ledermann JA, Kohn EC. PARP Inhibitors for BRCA1/2 mutation-associated and BRCA-like malignancies. Ann Oncol. 2014;25(1):32–40. 10.1093/annonc/mdt384 PMC3868320 24225019

[51] Javle M, Shacham-Shmueli E, Xiao L, Varadhachary G, Halpern N, Fogelman D, et al. Olaparib monotherapy for previously treated pancreatic cancer with DNA damage repair genetic alterations other than germline BRCA variants: Findings from 2 phase 2 nonrandomized clinical trials. JAMA Oncol. 2021;7(5):693–9. 10.1001/jamaoncol.2021.0006 PMC7934074 33662100

[52] Pant S, Maitra A, Yap TA. PARP inhibition - opportunities in pancreatic cancer. Nat Rev Clin Oncol. 2019;16(10):595–6. 10.1038/s41571-019-0257-6 31332344

[53] Peng Y, Liao Q, Tan W, Peng C, Hu Z, Chen Y, et al. The deubiquitylating enzyme USP15 regulates homologous recombination repair and cancer cell response to PARP inhibitors. Nat Commun. 2019;10(1):1224. 10.1038/s41467-019-09232-8 PMC6420636 30874560

[54] Gani C, Coackley C, Kumareswaran R, Schutze C, Krause M, Zafarana G, et al. In vivo studies of the PARP inhibitor, AZD-2281, in combination with fractionated radiotherapy: An exploration of the therapeutic ratio. Radiother Oncol. 2015;116(3):486–94. 10.1016/j.radonc.2015.08.003 26277432

[55] Loap P, Loirat D, Berger F, Rodrigues M, Bazire L, Pierga JY, et al. Concurrent olaparib and radiotherapy in patients with triple-negative breast cancer: The phase 1 olaparib and radiation therapy for triple-negative breast cancer trial. JAMA Oncol. 2022;8(12):1802–8. 10.1001/jamaoncol.2022.5074 PMC9614672 36301572

[56] Sun Y, Wang J, Ma Y, Li J, Sun X, Zhao X, et al. Radiation induces NORAD expression to promote ESCC radiotherapy resistance via EEPD1/ATR/CHK1 signalling and by inhibiting pri-miR-199a1 processing and the exosomal transfer of miR-199a-5p. J Exp Clin Cancer Res. 2021;40(1):306. 10.1186/s13046-021-02084-5 PMC8479908 34587992

[57] Xie B, Wang S, Jiang N, Li JJ. Cyclin B1/CDK1-regulated mitochondrial bioenergetics in cell cycle progression and tumor resistance. Cancer Lett. 2019;443:56–66. 10.1016/j.canlet.2018.11.019 PMC6759061 30481564

[58] Du X, Li J, Luo X, Li R, Li F, Zhang Y, et al. Structure-activity relationships of WEE1 inhibitors: A review. Eur J Med Chem. 2020;203:112524. 10.1016/j.ejmech.2020.112524 32688199

[59] Cheng B, Pan W, Xing Y, Xiao Y, Chen J, Xu Z. Recent advances in DDR (DNA damage response) inhibitors for cancer therapy. Eur J Med Chem. 2022;230:114109. 10.1016/j.ejmech.2022.114109 35051747

[60] Cleary JM, Aguirre AJ, Shapiro GI, D’Andrea AD. Biomarker-guided development of DNA repair inhibitors. Mol Cell. 2020;78(6):1070–85. 10.1016/j.molcel.2020.04.035 PMC7316088 32459988

[61] Ghelli Luserna Di Rora A, Bocconcelli M, Ferrari A, Terragna C, Bruno S, Imbrogno E, et al. Synergism through WEE1 and CHK1 inhibition in acute lymphoblastic leukemia. Cancers (Basel). 2019;11(11):1654. 10.3390/cancers11111654 PMC6895917 31717700

[62] Larsen BD, Benada J, Yung PYK, Bell RAV, Pappas G, Urban V, et al. Cancer cells use self-inflicted DNA breaks to evade growth limits imposed by genotoxic stress. Science. 2022;376(6592):476–83. 10.1126/science.abi6378 35482866

[63] Min A, Im SA. PARP inhibitors as therapeutics: Beyond modulation of PARylation. Cancers (Basel). 2020;12(2):394. 10.3390/cancers12020394 PMC7072193 32046300

[64] Chandran EA, Kennedy I. Significant tumor response to the poly(ADP-ribose) polymerase inhibitor olaparib in heavily pretreated patient with ovarian carcinosarcoma harboring a germline RAD51D mutation. JCO Precis Oncol. 2018;2:1–4. 10.1200/PO.18.00253 35135167

[65] Ceccaldi R, Liu JC, Amunugama R, Hajdu I, Primack B, Petalcorin MI, et al. Homologous-recombination-deficient tumours are dependent on Poltheta-mediated repair. Nature. 2015;518(7538):258–62. 10.1038/nature14184 PMC4415602 25642963

[66] Arneth B. Tumor Microenvironment. Medicina (Kaunas). 2019;56(1):15. 10.3390/medicina56010015 PMC7023392 31906017

[67] Bhandari V, Li CH, Bristow RG, Boutros PC, Consortium P. Divergent mutational processes distinguish hypoxic and normoxic tumours. Nat Commun. 2020;11(1):737. 10.1038/s41467-019-14052-x PMC7002770 32024819

[68] Dhani N, Fyles A, Hedley D, Milosevic M. The clinical significance of hypoxia in human cancers. Semin Nucl Med. 2015;45(2):110–21. 10.1053/j.semnuclmed.2014.11.002 25704384

[69] Span PN, Bussink J. Biology of hypoxia. Semin Nucl Med. 2015;45(2):101–9. 10.1053/j.semnuclmed.2014.10.002 25704383

[70] Hu J, Li X, Yang L, Li H. Hypoxia, a key factor in the immune microenvironment. Biomed Pharmacother. 2022;151:113068. 10.1016/j.biopha.2022.113068 35676780

[71] Graham K, Unger E. Overcoming tumor hypoxia as a barrier to radiotherapy, chemotherapy and immunotherapy in cancer treatment. Int J Nanomedicine. 2018;13:6049–58. 10.2147/IJN.S140462 PMC6177375 30323592

[72] Colliez F, Gallez B, Jordan BF. Assessing tumor oxygenation for predicting outcome in radiation oncology: A review of studies correlating tumor hypoxic status and outcome in the preclinical and clinical settings. Front Oncol. 2017;7:10. 10.3389/fonc.2017.00010 PMC5263142 28180110

[73] Schaue D, McBride WH. Opportunities and challenges of radiotherapy for treating cancer. Nat Rev Clin Oncol. 2015;12(9):527–40. 10.1038/nrclinonc.2015.120 PMC8396062 26122185

[74] Telarovic I, Wenger RH, Pruschy M. Interfering with tumor hypoxia for radiotherapy optimization. J Exp Clin Cancer Res. 2021;40(1):197. 10.1186/s13046-021-02000-x PMC8215813 34154610

[75] Grogan M, Thomas GM, Melamed I, Wong FL, Pearcey RG, Joseph PK, et al. The importance of hemoglobin levels during radiotherapy for carcinoma of the cervix. Cancer. 1999;86(8):1528–36. 10.1002/(sici)1097-0142(19991015)86:8<1528::aid-cncr20>3.0.co;2-e 10526282

[76] Rockwell S. Use of a perfluorochemical emulsion to improve oxygenation in a solid tumor. Int J Radiat Oncol Biol Phys. 1985;11(1):97–103. 10.1016/0360-3016(85)90367-0 3967986

[77] Henk JM, Kunkler PB, Smith CW. Radiotherapy and hyperbaric oxygen in head and neck cancer. Final report of first controlled clinical trial. Lancet. 1977;2(8029):101–3. 10.1016/s0140-6736(77)90116-7 69191

[78] Ashton TM, Fokas E, Kunz-Schughart LA, Folkes LK, Anbalagan S, Huether M, et al. The anti-malarial atovaquone increases radiosensitivity by alleviating tumour hypoxia. Nat Commun. 2016;7:12308. 10.1038/ncomms12308 PMC4962491 27453292

[79] Skwarski M, McGowan DR, Belcher E, Di Chiara F, Stavroulias D, McCole M, et al. Mitochondrial inhibitor atovaquone increases tumor oxygenation and inhibits hypoxic gene expression in patients with non-small cell lung cancer. Clin Cancer Res. 2021;27(9):2459–69. 10.1158/1078-0432.CCR-20-4128 PMC7611473 33597271

[80] Benej M, Hong X, Vibhute S, Scott S, Wu J, Graves E, et al. Papaverine and its derivatives radiosensitize solid tumors by inhibiting mitochondrial metabolism. Proc Natl Acad Sci U S A. 2018;115(42):10756–61. 10.1073/pnas.1808945115 PMC6196495 30201710

[81] Infantino V, Santarsiero A, Convertini P, Todisco S, Iacobazzi V. Cancer cell metabolism in hypoxia: Role of HIF-1 as key regulator and therapeutic target. Int J Mol Sci. 2021;22(11):5703. 10.3390/ijms22115703 PMC8199012 34071836

[82] Luo Y, Li M, Zuo X, Basourakos SP, Zhang J, Zhao J, et al. Beta‑catenin nuclear translocation induced by HIF‑1alpha overexpression leads to the radioresistance of prostate cancer. Int J Oncol. 2018;52(6):1827–40. 10.3892/ijo.2018.4368 PMC5919719 29658569

[83] Fu Z, Chen D, Cheng H, Wang F. Hypoxia-inducible factor-1alpha protects cervical carcinoma cells from apoptosis induced by radiation via modulation of vascular endothelial growth factor and p53 under hypoxia. Med Sci Monit. 2015;21:318–25. 10.12659/MSM.893265 PMC4316866 25623525

[84] Liu J, Zhang J, Wang X, Li Y, Chen Y, Li K, et al. HIF-1 and NDRG2 contribute to hypoxia-induced radioresistance of cervical cancer Hela cells. Exp Cell Res. 2010;316(12):1985–93. 10.1016/j.yexcr.2010.02.028 20206160

[85] Suwa T, Kobayashi M, Shirai Y, Nam JM, Tabuchi Y, Takeda N, et al. SPINK1 as a plasma marker for tumor hypoxia and a therapeutic target for radiosensitization. JCI Insight. 2021;6(21):e148135. 10.1172/jci.insight.148135 PMC8663551 34747365

[86] Zou YM, Hu GY, Zhao XQ, Lu T, Zhu F, Yu SY, et al. Hypoxia-induced autophagy contributes to radioresistance via c-Jun-mediated Beclin1 expression in lung cancer cells. J Huazhong Univ Sci Technolog Med Sci. 2014;34(5):761–7. 10.1007/s11596-014-1349-2 25318890

[87] Zhong R, Xu H, Chen G, Zhao G, Gao Y, Liu X, et al. The role of hypoxia-inducible factor-1alpha in radiation-induced autophagic cell death in breast cancer cells. Tumour Biol. 2015;36(9):7077–83. 10.1007/s13277-015-3425-z 25874499

[88] Xin Y, Jiang F, Yang C, Yan Q, Guo W, Huang Q, et al. Role of autophagy in regulating the radiosensitivity of tumor cells. J Cancer Res Clin Oncol. 2017;143(11):2147–57. 10.1007/s00432-017-2487-2 PMC11819394 28786037

[89] Ondrej M, Cechakova L, Durisova K, Pejchal J, Tichy A. To live or let die: Unclear task of autophagy in the radiosensitization battle. Radiother Oncol. 2016;119(2):265–75. 10.1016/j.radonc.2016.02.028 26993419

[90] Onorati AV, Dyczynski M, Ojha R, Amaravadi RK. Targeting autophagy in cancer. Cancer. 2018;124(16):3307–18. 10.1002/cncr.31335 PMC6108917 29671878

[91] Sun Y, Xing X, Liu Q, Wang Z, Xin Y, Zhang P, et al. Hypoxia-induced autophagy reduces radiosensitivity by the HIF-1alpha/miR-210/Bcl-2 pathway in colon cancer cells. Int J Oncol. 2015;46(2):750–6. 10.3892/ijo.2014.2745 25385144

[92] Shen Y, Liu Y, Sun T, Yang W. LincRNA-p21 knockdown enhances radiosensitivity of hypoxic tumor cells by reducing autophagy through HIF-1/Akt/mTOR/P70S6K pathway. Exp Cell Res. 2017;358(2):188–98. 10.1016/j.yexcr.2017.06.016 28689810

[93] Anbalagan S, Pires IM, Blick C, Hill MA, Ferguson DJ, Chan DA, et al. Radiosensitization of renal cell carcinoma in vitro through the induction of autophagy. Radiother Oncol. 2012;103(3):388–93. 10.1016/j.radonc.2012.04.001 22551566

[94] Moeller BJ, Dreher MR, Rabbani ZN, Schroeder T, Cao Y, Li CY, et al. Pleiotropic effects of HIF-1 blockade on tumor radiosensitivity. Cancer Cell. 2005;8(2):99–110. 10.1016/j.ccr.2005.06.016 16098463

[95] Zhang X, Kon T, Wang H, Li F, Huang Q, Rabbani ZN, et al. Enhancement of hypoxia-induced tumor cell death in vitro and radiation therapy in vivo by use of small interfering RNA targeted to hypoxia-inducible factor-1alpha. Cancer Res. 2004;64(22):8139–42. 10.1158/0008-5472.CAN-03-2301 15548675

[96] Williams KJ, Telfer BA, Xenaki D, Sheridan MR, Desbaillets I, Peters HJ, et al. Enhanced response to radiotherapy in tumours deficient in the function of hypoxia-inducible factor-1. Radiother Oncol. 2005;75(1):89–98. 10.1016/j.radonc.2005.01.009 15878106

[97] Liao C, Zhang Q. Understanding the oxygen-sensing pathway and its therapeutic implications in diseases. Am J Pathol. 2020;190(8):1584–95. 10.1016/j.ajpath.2020.04.003 PMC7416076 32339495

[98] Zighelboim I, Wright JD, Gao F, Case AS, Massad LS, Mutch DG, et al. Multicenter phase II trial of topotecan, cisplatin and bevacizumab for recurrent or persistent cervical cancer. Gynecol Oncol. 2013;130(1):64–8. 10.1016/j.ygyno.2013.04.009 PMC3870479 23591400

[99] Subramaniam DS, Liu SV, Crawford J, Kramer J, Thompson J, Wang H, et al. A phase Ib/II study of ganetespib with doxorubicin in advanced solid tumors including relapsed-refractory small cell lung cancer. Front Oncol. 2018;8:64. 10.3389/fonc.2018.00064 PMC5858550 29594044

[100] Garrett CR, Bekaii-Saab TS, Ryan T, Fisher GA, Clive S, Kavan P, et al. Randomized phase 2 study of pegylated SN-38 (EZN-2208) or irinotecan plus cetuximab in patients with advanced colorectal cancer. Cancer. 2013;119(24):4223–30. 10.1002/cncr.28358 24105075

[101] Jeong W, Rapisarda A, Park SR, Kinders RJ, Chen A, Melillo G, et al. Pilot trial of EZN-2968, an antisense oligonucleotide inhibitor of hypoxia-inducible factor-1 alpha (HIF-1alpha), in patients with refractory solid tumors. Cancer Chemother Pharmacol. 2014;73(2):343–8. 10.1007/s00280-013-2362-z PMC8375568 24292632

[102] Kao TW, Bai GH, Wang TL, Shih IM, Chuang CM, Lo CL, et al. Novel cancer treatment paradigm targeting hypoxia-induced factor in conjunction with current therapies to overcome resistance. J Exp Clin Cancer Res. 2023;42(1):171. 10.1186/s13046-023-02724-y PMC10353250 37460927

[103] Vaupel P, Schmidberger H, Mayer A. The Warburg effect: essential part of metabolic reprogramming and central contributor to cancer progression. Int J Radiat Biol. 2019;95(7):912–9. 10.1080/09553002.2019.1589653 30822194

[104] Al Tameemi W, Dale TP, Al-Jumaily RMK, Forsyth NR. Hypoxia-modified cancer cell metabolism. Front Cell Dev Biol. 2019;7:4. 10.3389/fcell.2019.00004 PMC6362613 30761299

[105] Dalzoppo D, Di Paolo V, Calderan L, Pasut G, Rosato A, Caccuri AM, et al. Thiol-activated anticancer agents: The state of the art. Anticancer Agents Med Chem. 2017;17(1):4–20. 27539318

[106] Vaupel P, Multhoff G. Fatal alliance of hypoxia-/HIF-1alpha-driven microenvironmental traits promoting cancer progression. Adv Exp Med Biol. 2020;1232:169–76. 10.1007/978-3-030-34461-0_21 31893407

[107] Leung E, Cairns RA, Chaudary N, Vellanki RN, Kalliomaki T, Moriyama EH, et al. Metabolic targeting of HIF-dependent glycolysis reduces lactate, increases oxygen consumption and enhances response to high-dose single-fraction radiotherapy in hypoxic solid tumors. BMC Cancer. 2017;17(1):418. 10.1186/s12885-017-3402-6 PMC5473006 28619042

[108] McDonald PC, Chafe SC, Brown WS, Saberi S, Swayampakula M, Venkateswaran G, et al. Regulation of pH by carbonic anhydrase 9 mediates survival of pancreatic cancer cells with activated KRAS in response to hypoxia. Gastroenterology. 2019;157(3):823–37. 10.1053/j.gastro.2019.05.004 31078621

[109] Shen H, Hau E, Joshi S, Dilda PJ, McDonald KL. Sensitization of glioblastoma cells to irradiation by modulating the glucose metabolism. Mol Cancer Ther. 2015;14(8):1794–804. 10.1158/1535-7163.MCT-15-0247 26063767

[110] Heller S, Maurer GD, Wanka C, Hofmann U, Luger AL, Bruns I, et al. Gene suppression of transketolase-like protein 1 (TKTL1) sensitizes glioma cells to hypoxia and ionizing radiation. Int J Mol Sci. 2018;19(8):2168. 10.3390/ijms19082168 PMC6121283 30044385

[111] Chae YC, Angelin A, Lisanti S, Kossenkov AV, Speicher KD, Wang H, et al. Corrigendum: Landscape of the mitochondrial Hsp90 metabolome in tumours. Nat Commun. 2015;6:7605. 10.1038/ncomms8605 PMC4750482 26085380

[112] Matassa DS, Agliarulo I, Avolio R, Landriscina M, Esposito F. TRAP1 regulation of cancer metabolism: Dual Role as oncogene or tumor suppressor. Genes (Basel). 2018;9(4):195. 10.3390/genes9040195 PMC5924537 29621137

[113] Avolio R, Matassa DS, Criscuolo D, Landriscina M, Esposito F. Modulation of mitochondrial metabolic reprogramming and oxidative stress to overcome chemoresistance in cancer. Biomolecules. 2020;10(1):135. 10.3390/biom10010135 PMC7023176 31947673

[114] Chhipa RR, Fan Q, Anderson J, Muraleedharan R, Huang Y, Ciraolo G, et al. AMP kinase promotes glioblastoma bioenergetics and tumour growth. Nat Cell Biol. 2018;20(7):823–35. 10.1038/s41556-018-0126-z PMC6113057 29915361

[115] Cao K, Li J, Chen J, Qian L, Wang A, Chen X, et al. microRNA-33a-5p increases radiosensitivity by inhibiting glycolysis in melanoma. Oncotarget. 2017;8(48):83660–72. 10.18632/oncotarget.19014 PMC5663544 29137372

[116] Dai C. The heat-shock, or HSF1-mediated proteotoxic stress, response in cancer: from proteomic stability to oncogenesis. Philos Trans R Soc Lond B Biol Sci. 2018;373(1738):20160525. 10.1098/rstb.2016.0525 PMC5717525 29203710

[117] Li Q, Martinez JD. Loss of HSF1 results in defective radiation-induced G(2) arrest and DNA repair. Radiat Res. 2011;176(1):17–24. 10.1667/rr2393.1 PMC3142266 21557666

[118] Kabakov A, Yakimova A, Matchuk O. Molecular chaperones in cancer stem cells: Determinants of stemness and potential targets for antitumor therapy. Cells. 2020;9(4):892. 10.3390/cells9040892 PMC7226806 32268506

[119] Vilaboa NE, Galan A, Troyano A, de Blas E, Aller P. Regulation of multidrug resistance 1 (MDR1)/P-glycoprotein gene expression and activity by heat-shock transcription factor 1 (HSF1). J Biol Chem. 2000;275(32):24970–6. 10.1074/jbc.M909136199 10816597

[120] Prager BC, Xie Q, Bao S, Rich JN. Cancer stem cells: The architects of the tumor ecosystem. Cell Stem Cell. 2019;24(1):41–53. 10.1016/j.stem.2018.12.009 PMC6350931 30609398

[121] Bamodu OA, Chang HL, Ong JR, Lee WH, Yeh CT, Tsai JT. Elevated PDK1 expression drives PI3K/AKT/MTOR signaling promotes radiation-resistant and dedifferentiated phenotype of hepatocellular carcinoma. Cells. 2020;9(3):746. 10.3390/cells9030746 PMC7140693 32197467

[122] Yang L, Shi P, Zhao G, Xu J, Peng W, Zhang J, et al. Targeting cancer stem cell pathways for cancer therapy. Signal Transduct Target Ther. 2020;5(1):8. 10.1038/s41392-020-0110-5 PMC7005297 32296030

[123] Zhao H, Jiang H, Li Z, Zhuang Y, Liu Y, Zhou S, et al. 2-Methoxyestradiol enhances radiosensitivity in radioresistant melanoma MDA-MB-435R cells by regulating glycolysis via HIF-1alpha/PDK1 axis. Int J Oncol. 2017;50(5):1531–40. 10.3892/ijo.2017.3924 PMC5403226 28339028

[124] Amaresan R, Gopal U. Cell surface GRP78: a potential mechanism of therapeutic resistant tumors. Cancer Cell Int. 2023;23(1):100. 10.1186/s12935-023-02931-9 PMC10204160 37221596

[125] Jiang CC, Marsland M, Wang Y, Dowdell A, Eden E, Gao F, et al. Tumor innervation is triggered by endoplasmic reticulum stress. Oncogene. 2022;41(4):586–99. 10.1038/s41388-021-02108-6 34785777

[126] Hua Y, Huang JH, Shao ZH, Luo XM, Wang ZY, Liu JQ, et al. Composition-dependent enzyme mimicking activity and radiosensitizing effect of bimetallic clusters to modulate tumor hypoxia for enhanced cancer therapy. Adv Mater. 2022;34(31):e2203734. 10.1002/adma.202203734 35681250

[127] Zai W, Kang L, Dong T, Wang H, Yin L, Gan S, et al. E. coli membrane vesicles as a catalase carrier for long-term tumor hypoxia relief to enhance radiotherapy. ACS Nano. 2021;15(9):15381–94. 10.1021/acsnano.1c07621 34520168

[128] Yao X, Lu S, Feng C, Suo R, Li H, Zhang Y, et al. Tumor oxygenation nanoliposome synergistic hypoxia-inducible-factor-1 inhibitor enhanced Iodine-125 seed brachytherapy for esophageal cancer. Biomaterials. 2022;289:121801. 10.1016/j.biomaterials.2022.121801 36137416

[129] Chai R, Yu L, Dong C, Yin Y, Wang S, Chen Y, et al. Oxygen-evolving photosynthetic cyanobacteria for 2D bismuthene radiosensitizer-enhanced cancer radiotherapy. Bioact Mater. 2022;17:276–88. 10.1016/j.bioactmat.2022.01.014 PMC8965086 35386463

[130] O’Brien CA, Kreso A, Dick JE. Cancer stem cells in solid tumors: an overview. Semin Radiat Oncol. 2009;19(2):71–7. 10.1016/j.semradonc.2008.11.001 19249644

[131] Batlle E, Clevers H. Cancer stem cells revisited. Nat Med. 2017;23(10):1124–34. 10.1038/nm.4409 28985214

[132] Peitzsch C, Kurth I, Kunz-Schughart L, Baumann M, Dubrovska A. Discovery of the cancer stem cell related determinants of radioresistance. Radiother Oncol. 2013;108(3):378–87. 10.1016/j.radonc.2013.06.003 23830195

[133] Driessens G, Beck B, Caauwe A, Simons BD, Blanpain C. Defining the mode of tumour growth by clonal analysis. Nature. 2012;488(7412):527–30. 10.1038/nature11344 PMC5553110 22854777

[134] Schulz A, Meyer F, Dubrovska A, Borgmann K. Cancer stem cells and radioresistance: DNA repair and beyond. Cancers (Basel). 2019;11(6):862. 10.3390/cancers11060862 PMC6627210 31234336

[135] Dhanasekaran R, Deutzmann A, Mahauad-Fernandez WD, Hansen AS, Gouw AM, Felsher DW. The MYC oncogene - the grand orchestrator of cancer growth and immune evasion. Nat Rev Clin Oncol. 2022;19(1):23–36. 10.1038/s41571-021-00549-2 PMC9083341 34508258

[136] Wang WJ, Wu SP, Liu JB, Shi YS, Huang X, Zhang QB, et al. MYC regulation of CHK1 and CHK2 promotes radioresistance in a stem cell-like population of nasopharyngeal carcinoma cells. Cancer Res. 2013;73(3):1219–31. 10.1158/0008-5472.CAN-12-1408 23269272

[137] Nathansen J, Lukiyanchuk V, Hein L, Stolte MI, Borgmann K, Lock S, et al. Oct4 confers stemness and radioresistance to head and neck squamous cell carcinoma by regulating the homologous recombination factors PSMC3IP and RAD54L. Oncogene. 2021;40(24):4214–28. 10.1038/s41388-021-01842-1 PMC8211562 34079088

[138] Zhang S, Xiong X, Sun Y. Functional characterization of SOX2 as an anticancer target. Signal Transduct Target Ther. 2020;5(1):135. 10.1038/s41392-020-00242-3 PMC7391717 32728033

[139] Huang C, Lu H, Li J, Xie X, Fan L, Wang D, et al. SOX2 regulates radioresistance in cervical cancer via the hedgehog signaling pathway. Gynecol Oncol. 2018;151(3):533–41. 10.1016/j.ygyno.2018.10.005 30336948

[140] Bai X, Ni J, Beretov J, Wang S, Dong X, Graham P, et al. THOC2 and THOC5 regulate stemness and radioresistance in triple-negative breast cancer. Adv Sci (Weinh). 2021;8(24):e2102658. 10.1002/advs.202102658 PMC8693071 34708581

[141] de Araujo PR, Gorthi A, da Silva AE, Tonapi SS, Vo DT, Burns SC, et al. Musashi1 impacts radio-resistance in glioblastoma by controlling DNA-protein kinase catalytic subunit. Am J Pathol. 2016;186(9):2271–8. 10.1016/j.ajpath.2016.05.020 PMC5012509 27470713

[142] Lee JK, Chang N, Yoon Y, Yang H, Cho H, Kim E, et al. USP1 targeting impedes GBM growth by inhibiting stem cell maintenance and radioresistance. Neuro Oncol. 2016;18(1):37–47. 10.1093/neuonc/nov091 PMC4677407 26032834

[143] Park SY, Lee CJ, Choi JH, Kim JH, Kim JW, Kim JY, et al. The JAK2/STAT3/CCND2 Axis promotes colorectal Cancer stem cell persistence and radioresistance. J Exp Clin Cancer Res. 2019;38(1):399. 10.1186/s13046-019-1405-7 PMC6737692 31511084

[144] Chang L, Graham PH, Hao J, Ni J, Bucci J, Cozzi PJ, et al. Acquisition of epithelial-mesenchymal transition and cancer stem cell phenotypes is associated with activation of the PI3K/Akt/mTOR pathway in prostate cancer radioresistance. Cell Death Dis. 2013;4(10):e875. 10.1038/cddis.2013.407 PMC3920940 24157869

[145] Liu Z, Wu K, Gu S, Wang W, Xie S, Lu T, et al. A methyltransferase-like 14/miR-99a-5p/tribble 2 positive feedback circuit promotes cancer stem cell persistence and radioresistance via histone deacetylase 2-mediated epigenetic modulation in esophageal squamous cell carcinoma. Clin Transl Med. 2021;11(9):e545. 10.1002/ctm2.545 PMC8441142 34586732

[146] Sun X, He Z, Guo L, Wang C, Lin C, Ye L, et al. ALG3 contributes to stemness and radioresistance through regulating glycosylation of TGF-beta receptor II in breast cancer. J Exp Clin Cancer Res. 2021;40(1):149. 10.1186/s13046-021-01932-8 PMC8086123 33931075

[147] Wang C, Liu L, Cheng Y, Shi H. Combined GSK-3beta and MEK inhibitors modulate the stemness and radiotherapy sensitivity of cervical cancer stem cells through the WNT signaling pathway. Chem Biol Interact. 2023;380:110515. 10.1016/j.cbi.2023.110515 37116855

[148] Chi HC, Tsai CY, Wang CS, Yang HY, Lo CH, Wang WJ, et al. DOCK6 promotes chemo- and radioresistance of gastric cancer by modulating WNT/beta-catenin signaling and cancer stem cell traits. Oncogene. 2020;39(37):5933–49. 10.1038/s41388-020-01390-0 32753649

[149] Sun T, Yin YF, Jin HG, Liu HR, Tian WC. Exosomal microRNA-19b targets FBXW7 to promote colorectal cancer stem cell stemness and induce resistance to radiotherapy. Kaohsiung J Med Sci. 2022;38(2):108–19. 10.1002/kjm2.12449 PMC11896196 34520626

[150] Yang M, Liu Q, Dai M, Peng R, Li X, Zuo W, et al. FOXQ1-mediated SIRT1 upregulation enhances stemness and radio-resistance of colorectal cancer cells and restores intestinal microbiota function by promoting beta-catenin nuclear translocation. J Exp Clin Cancer Res. 2022;41(1):70. 10.1186/s13046-021-02239-4 PMC8857837 35183223

[151] Lu Y, Liang Y, Zheng X, Deng X, Huang W, Zhang G. EVI1 promotes epithelial-to-mesenchymal transition, cancer stem cell features and chemo-/radioresistance in nasopharyngeal carcinoma. J Exp Clin Cancer Res. 2019;38(1):82. 10.1186/s13046-019-1077-3 PMC6377731 30770775

[152] Kang H, Lee S, Kim K, Jeon J, Kang SG, Youn H, et al. Downregulated CLIP3 induces radioresistance by enhancing stemness and glycolytic flux in glioblastoma. J Exp Clin Cancer Res. 2021;40(1):282. 10.1186/s13046-021-02077-4 PMC8420000 34488821

[153] Wang Y, Zhang L, Sun XL, Lu YC, Chen S, Pei DS, et al. NRP1 contributes to stemness and potentiates radioresistance via WTAP-mediated m6A methylation of Bcl-2 mRNA in breast cancer. Apoptosis. 2023;28(1–2):233–46. 10.1007/s10495-022-01784-3 36333630

[154] Park SJ, Min HJ, Yoon C, Kim SH, Kim JH, Lee SY. Integrin beta1 regulates the perineural invasion and radioresistance of oral squamous carcinoma cells by modulating cancer cell stemness. Cell Signal. 2023;110:110808. 10.1016/j.cellsig.2023.110808 37481218

[155] Chen E, Wang T, Tu Y, Sun Z, Ding Y, Gu Z, et al. ROS-scavenging biomaterials for periodontitis. J Mater Chem B. 2023;11(3):482–99. 10.1039/d2tb02319a 36468674

[156] Wang Y, Qi H, Liu Y, Duan C, Liu X, Xia T, et al. The double-edged roles of ROS in cancer prevention and therapy. Theranostics. 2021;11(10):4839–57. 10.7150/thno.56747 PMC7978298 33754031

[157] Zhan Y, Zhang Z, Liu Y, Fang Y, Xie Y, Zheng Y, et al. NUPR1 contributes to radiation resistance by maintaining ROS homeostasis via AhR/CYP signal axis in hepatocellular carcinoma. BMC Med. 2022;20(1):365. 10.1186/s12916-022-02554-3 PMC9580158 36258210

[158] Cojoc M, Mabert K, Muders MH, Dubrovska A. A role for cancer stem cells in therapy resistance: cellular and molecular mechanisms. Semin Cancer Biol. 2015;31:16–27. 10.1016/j.semcancer.2014.06.004 24956577

[159] Johnson A, Iffland-Muhlhaus L, Northcote-Smith J, Singh K, Ortu F, Apfel UP, et al. A bioinspired redox-modulating copper(II)-macrocyclic complex bearing non-steroidal anti-inflammatory drugs with anti-cancer stem cell activity. Dalton Trans. 2022;51(15):5904–12.10.1039/d2dt00788f35348171

[160] Mukha A, Kahya U, Linge A, Chen O, Lock S, Lukiyanchuk V, et al. GLS-driven glutamine catabolism contributes to prostate cancer radiosensitivity by regulating the redox state, stemness and ATG5-mediated autophagy. Theranostics. 2021;11(16):7844–68. 10.7150/thno.58655 PMC8315064 34335968

[161] Praharaj PP, Singh A, Patra S, Bhutia SK. Co-targeting autophagy and NRF2 signaling triggers mitochondrial superoxide to sensitize oral cancer stem cells for cisplatin-induced apoptosis. Free Radic Biol Med. 2023;207:72–88. 10.1016/j.freeradbiomed.2023.07.008 37423560

[162] Chen Y, Li D, Wang D, Liu X, Yin N, Song Y, et al. Quiescence and attenuated DNA damage response promote survival of esophageal cancer stem cells. J Cell Biochem. 2012;113(12):3643–52. 10.1002/jcb.24228 22711554

[163] Tsai CY, Ko HJ, Huang CF, Lin CY, Chiou SJ, Su YF, et al. Ionizing radiation induces resistant glioblastoma stem-like cells by promoting autophagy via the Wnt/beta-catenin pathway. Life (Basel). 2021;11(5):451. 10.3390/life11050451 PMC8157563 34069945

[164] Rothe K, Porter V, Jiang X. Current outlook on autophagy in human leukemia: Foe in cancer stem cells and drug resistance, friend in new therapeutic interventions. Int J Mol Sci. 2019;20(3):461. 10.3390/ijms20030461 PMC6387281 30678185

[165] Digomann D, Linge A, Dubrovska A. SLC3A2/CD98hc, autophagy and tumor radioresistance: a link confirmed. Autophagy. 2019;15(10):1850–1. 10.1080/15548627.2019.1639302 PMC6735542 31276435

[166] Mohyeldin A, Garzon-Muvdi T, Quinones-Hinojosa A. Oxygen in stem cell biology: a critical component of the stem cell niche. Cell Stem Cell. 2010;7(2):150–61. 10.1016/j.stem.2010.07.007 20682444

[167] Peitzsch C, Perrin R, Hill RP, Dubrovska A, Kurth I. Hypoxia as a biomarker for radioresistant cancer stem cells. Int J Radiat Biol. 2014;90(8):636–52. 10.3109/09553002.2014.916841 24844374

[168] Schoning JP, Monteiro M, Gu W. Drug resistance and cancer stem cells: the shared but distinct roles of hypoxia-inducible factors HIF1alpha and HIF2alpha. Clin Exp Pharmacol Physiol. 2017;44(2):153–61. 10.1111/1440-1681.12693 27809360

[169] Weinberg F, Ramnath N, Nagrath D. Reactive Oxygen species in the tumor microenvironment: An overview. Cancers (Basel). 2019;11(8):1191. 10.3390/cancers11081191 PMC6721577 31426364

[170] Zhu Y, Wang C, Becker SA, Hurst K, Nogueira LM, Findlay VJ, et al. miR-145 antagonizes SNAI1-mediated stemness and radiation resistance in colorectal cancer. Mol Ther. 2018;26(3):744–54. 10.1016/j.ymthe.2017.12.023 PMC5910672 29475734

[171] Lin C, Verma V, Ly QP, Lazenby A, Sasson A, Schwarz JK, et al. Phase I trial of concurrent stereotactic body radiotherapy and nelfinavir for locally advanced borderline or unresectable pancreatic adenocarcinoma. Radiother Oncol. 2019;132:55–62. 10.1016/j.radonc.2018.11.002 PMC6400311 30825970

[172] Golden EB, Chhabra A, Chachoua A, Adams S, Donach M, Fenton-Kerimian M, et al. Local radiotherapy and granulocyte-macrophage colony-stimulating factor to generate abscopal responses in patients with metastatic solid tumours: a proof-of-principle trial. Lancet Oncol. 2015;16(7):795–803. 10.1016/S1470-2045(15)00054-6 26095785

[173] Li MO, Wolf N, Raulet DH, Akkari L, Pittet MJ, Rodriguez PC, et al. Innate immune cells in the tumor microenvironment. Cancer Cell. 2021;39(6):725–9. 10.1016/j.ccell.2021.05.016 34129817

[174] Galluzzi L, Vanpouille-Box C, Bakhoum SF, Demaria S. SnapShot: CGAS-STING Signaling. Cell. 2018;173(1):276-e1. 10.1016/j.cell.2018.03.015 29570996

[175] Zhao Y, Liu Z, Liu G, Zhang Y, Liu S, Gan D, et al. Neutrophils resist ferroptosis and promote breast cancer metastasis through aconitate decarboxylase 1. Cell Metab. 2023;35(10):1688–703 e10. 10.1016/j.cmet.2023.09.004 PMC10558089 37793345

[176] De Martino M, Daviaud C, Diamond JM, Kraynak J, Alard A, Formenti SC, et al. Activin a promotes regulatory T-cell-mediated immunosuppression in irradiated breast cancer. Cancer Immunol Res. 2021;9(1):89–102. 10.1158/2326-6066.CIR-19-0305 PMC7785684 33093219

[177] Faget DV, Ren Q, Stewart SA. Unmasking senescence: context-dependent effects of SASP in cancer. Nat Rev Cancer. 2019;19(8):439–53. 10.1038/s41568-019-0156-2 31235879

[178] Rodriguez-Ruiz ME, Vitale I, Harrington KJ, Melero I, Galluzzi L. Immunological impact of cell death signaling driven by radiation on the tumor microenvironment. Nat Immunol. 2020;21(2):120–34. 10.1038/s41590-019-0561-4 31873291

[179] Gil del Alcazar CR, Hardebeck MC, Mukherjee B, Tomimatsu N, Gao X, Yan J, et al. Inhibition of DNA double-strand break repair by the dual PI3K/mTOR inhibitor NVP-BEZ235 as a strategy for radiosensitization of glioblastoma. Clin Cancer Res. 2014;20(5):1235–48. 10.1158/1078-0432.CCR-13-1607 PMC3947495 24366691

[180] Dong C, He M, Tu W, Konishi T, Liu W, Xie Y, et al. The differential role of human macrophage in triggering secondary bystander effects after either gamma-ray or carbon beam irradiation. Cancer Lett. 2015;363(1):92–100. 10.1016/j.canlet.2015.04.013 PMC4428931 25896631

[181] Dong S, Liang S, Cheng Z, Zhang X, Luo L, Li L, et al. ROS/PI3K/Akt and Wnt/beta-catenin signalings activate HIF-1alpha-induced metabolic reprogramming to impart 5-fluorouracil resistance in colorectal cancer. J Exp Clin Cancer Res. 2022;41(1):15. 10.1186/s13046-021-02229-6 PMC8742403 34998404

[182] Zhang T, Zhu X, Wu H, Jiang K, Zhao G, Shaukat A, et al. Targeting the ROS/PI3K/AKT/HIF-1alpha/HK2 axis of breast cancer cells: Combined administration of Polydatin and 2-Deoxy-d-glucose. J Cell Mol Med. 2019;23(5):3711–23. 10.1111/jcmm.14276 PMC6484306 30920152

[183] Yang J, Pi C, Wang G. Inhibition of PI3K/Akt/mTOR pathway by apigenin induces apoptosis and autophagy in hepatocellular carcinoma cells. Biomed Pharmacother. 2018;103:699–707. 10.1016/j.biopha.2018.04.072 29680738

[184] Wang NH, Lei Z, Yang HN, Tang Z, Yang MQ, Wang Y, et al. Radiation-induced PD-L1 expression in tumor and its microenvironment facilitates cancer-immune escape: a narrative review. Ann Transl Med. 2022;10(24):1406. 10.21037/atm-22-6049 PMC9843429 36660640

[185] Du SS, Chen GW, Yang P, Chen YX, Hu Y, Zhao QQ, et al. Radiation therapy promotes hepatocellular carcinoma immune cloaking via PD-L1 upregulation induced by cGAS-STING activation. Int J Radiat Oncol Biol Phys. 2022;112(5):1243–55. 10.1016/j.ijrobp.2021.12.162 34986380

[186] Deng L, Liang H, Burnette B, Beckett M, Darga T, Weichselbaum RR, et al. Irradiation and anti-PD-L1 treatment synergistically promote antitumor immunity in mice. J Clin Invest. 2014;124(2):687–95. 10.1172/JCI67313 PMC3904601 24382348

[187] Yi M, Zheng X, Niu M, Zhu S, Ge H, Wu K. Combination strategies with PD-1/PD-L1 blockade: current advances and future directions. Mol Cancer. 2022;21(1):28. 10.1186/s12943-021-01489-2 PMC8780712 35062949

[188] Olson DJ, Eroglu Z, Brockstein B, Poklepovic AS, Bajaj M, Babu S, et al. Pembrolizumab plus ipilimumab following anti-PD-1/L1 failure in melanoma. J Clin Oncol. 2021;39(24):2647–55. 10.1200/JCO.21.00079 PMC8376314 33945288

[189] Li C, Zhao S, Zheng Y, Han Y, Chen X, Cheng Z, et al. Preoperative pembrolizumab combined with chemoradiotherapy for oesophageal squamous cell carcinoma (PALACE-1). Eur J Cancer. 2021;144:232–41. 10.1016/j.ejca.2020.11.039 33373868

[190] Theelen W, Peulen HMU, Lalezari F, van der Noort V, de Vries JF, Aerts J, et al. Effect of pembrolizumab after stereotactic body radiotherapy vs pembrolizumab alone on tumor response in patients with advanced non-small cell lung cancer: Results of the PEMBRO-RT phase 2 randomized clinical trial. JAMA Oncol. 2019;5(9):1276–82. 10.1001/jamaoncol.2019.1478 PMC6624814 31294749

[191] Theelen W, Chen D, Verma V, Hobbs BP, Peulen HMU, Aerts J, et al. Pembrolizumab with or without radiotherapy for metastatic non-small-cell lung cancer: a pooled analysis of two randomised trials. Lancet Respir Med. 2021;9(5):467–75. 10.1016/S2213-2600(20)30391-X 33096027

[192] Tao Y, Biau J, Sun XS, Sire C, Martin L, Alfonsi M, et al. Pembrolizumab versus cetuximab concurrent with radiotherapy in patients with locally advanced squamous cell carcinoma of head and neck unfit for cisplatin (GORTEC 2015-01 PembroRad): a multicenter, randomized, phase II trial. Ann Oncol. 2023;34(1):101–10. 10.1016/j.annonc.2022.10.006 36522816

[193] Twyman-Saint Victor C, Rech AJ, Maity A, Rengan R, Pauken KE, Stelekati E, et al. Radiation and dual checkpoint blockade activate non-redundant immune mechanisms in cancer. Nature. 2015;520(7547):373–7. 10.1038/nature14292 PMC4401634 25754329

[194] Formenti SC, Rudqvist NP, Golden E, Cooper B, Wennerberg E, Lhuillier C, et al. Radiotherapy induces responses of lung cancer to CTLA-4 blockade. Nat Med. 2018;24(12):1845–51. 10.1038/s41591-018-0232-2 PMC6286242 30397353

[195] Li JY, Zhao Y, Gong S, Wang MM, Liu X, He QM, et al. TRIM21 inhibits irradiation-induced mitochondrial DNA release and impairs antitumour immunity in nasopharyngeal carcinoma tumour models. Nat Commun. 2023;14(1):865. 10.1038/s41467-023-36523-y PMC9935546 36797289

[196] Houthuijzen JM, Jonkers J. Cancer-associated fibroblasts as key regulators of the breast cancer tumor microenvironment. Cancer Metastasis Rev. 2018;37(4):577–97. 10.1007/s10555-018-9768-3 30465162

[197] Liu L, Zhang Z, Zhou L, Hu L, Yin C, Qing D, et al. Cancer associated fibroblasts-derived exosomes contribute to radioresistance through promoting colorectal cancer stem cells phenotype. Exp Cell Res. 2020;391(2):111956. 10.1016/j.yexcr.2020.111956 32169425

[198] Mantoni TS, Lunardi S, Al-Assar O, Masamune A, Brunner TB. Pancreatic stellate cells radioprotect pancreatic cancer cells through beta1-integrin signaling. Cancer Res. 2011;71(10):3453–8. 10.1158/0008-5472.CAN-10-1633 PMC3097171 21558392

[199] Al-Assar O, Demiciorglu F, Lunardi S, Gaspar-Carvalho MM, McKenna WG, Muschel RM, et al. Contextual regulation of pancreatic cancer stem cell phenotype and radioresistance by pancreatic stellate cells. Radiother Oncol. 2014;111(2):243–51. 10.1016/j.radonc.2014.03.014 24780634

[200] Chen X, Liu J, Zhang Q, Liu B, Cheng Y, Zhang Y, et al. Exosome-mediated transfer of miR-93-5p from cancer-associated fibroblasts confer radioresistance in colorectal cancer cells by downregulating FOXA1 and upregulating TGFB3. J Exp Clin Cancer Res. 2020;39(1):65. 10.1186/s13046-019-1507-2 PMC7158087 32293494

[201] Zhang H, Yue J, Jiang Z, Zhou R, Xie R, Xu Y, et al. CAF-secreted CXCL1 conferred radioresistance by regulating DNA damage response in a ROS-dependent manner in esophageal squamous cell carcinoma. Cell Death Dis. 2017;8(5):e2790. 10.1038/cddis.2017.180 PMC5520705 28518141

[202] Huang W, Zhang L, Yang M, Wu X, Wang X, Huang W, et al. Cancer-associated fibroblasts promote the survival of irradiated nasopharyngeal carcinoma cells via the NF-kappaB pathway. J Exp Clin Cancer Res. 2021;40(1):87. 10.1186/s13046-021-01878-x PMC7923322 33648530

[203] Boelens MC, Wu TJ, Nabet BY, Xu B, Qiu Y, Yoon T, et al. Exosome transfer from stromal to breast cancer cells regulates therapy resistance pathways. Cell. 2014;159(3):499–513. 10.1016/j.cell.2014.09.051 PMC4283810 25417103

[204] Zhang R, Qi F, Zhao F, Li G, Shao S, Zhang X, et al. Cancer-associated fibroblasts enhance tumor-associated macrophages enrichment and suppress NK cells function in colorectal cancer. Cell Death Dis. 2019;10(4):273. 10.1038/s41419-019-1435-2 PMC6426970 30894509

[205] Mao X, Xu J, Wang W, Liang C, Hua J, Liu J, et al. Crosstalk between cancer-associated fibroblasts and immune cells in the tumor microenvironment: new findings and future perspectives. Mol Cancer. 2021;20(1):131. 10.1186/s12943-021-01428-1 PMC8504100 34635121

[206] Ogawa K, Murayama S, Mori M. Predicting the tumor response to radiotherapy using microarray analysis (Review). Oncol Rep. 2007;18(5):1243–8. 17914580

[207] Chen Y, Huang M, Yan Y, He D. Tranilast inhibits angiotensin II-induced myocardial fibrosis through S100A11/transforming growth factor-beta (TGF-beta1)/Smad axis. Bioengineered. 2021;12(1):8447–56. 10.1080/21655979.2021.1982322 PMC8806955 34663163

[208] Massoud G, Parish M, Hazimeh D, Moukarzel P, Singh B, Cayton Vaught KC, et al. Unlocking the potential of tranilast: Targeting fibrotic signaling pathways for therapeutic benefit. Int Immunopharmacol. 2024;137:112423. 10.1016/j.intimp.2024.112423 PMC11245748 38861914

[209] Ochi K, Suzawa K, Thu YM, Takatsu F, Tsudaka S, Zhu Y, et al. Drug repositioning of tranilast to sensitize a cancer therapy by targeting cancer-associated fibroblast. Cancer Sci. 2022;113(10):3428–36. 10.1111/cas.15502 PMC9530873 35871750

[210] Toh TB, Lim JJ, Chow EK. Epigenetics in cancer stem cells. Mol Cancer. 2017;16(1):29. 10.1186/s12943-017-0596-9 PMC5286794 28148257

[211] Nebbioso A, Tambaro FP, Dell’Aversana C, Altucci L. Cancer epigenetics: Moving forward. PLoS Genet. 2018;14(6):e1007362. 10.1371/journal.pgen.1007362 PMC5991666 29879107

[212] Miousse IR, Kutanzi KR, Koturbash I. Effects of ionizing radiation on DNA methylation: from experimental biology to clinical applications. Int J Radiat Biol. 2017;93(5):457–69. 10.1080/09553002.2017.1287454 PMC5411327 28134023

[213] Daino K, Nishimura M, Imaoka T, Takabatake M, Morioka T, Nishimura Y, et al. Epigenetic dysregulation of key developmental genes in radiation-induced rat mammary carcinomas. Int J Cancer. 2018;143(2):343–54. 10.1002/ijc.31309 29435983

[214] Li G, Luo R, Zhang W, He S, Wang B, Liang H, et al. m6A hypomethylation of DNMT3B regulated by ALKBH5 promotes intervertebral disc degeneration via E4F1 deficiency. Clin Transl Med. 2022;12(3):e765. 10.1002/ctm2.765 PMC8957938 35340126

[215] Wu C, Guo E, Ming J, Sun W, Nie X, Sun L, et al. Radiation-induced DNMT3B promotes radioresistance in nasopharyngeal carcinoma through methylation of p53 and p21. Mol Ther Oncolytics. 2020;17:306–19. 10.1016/j.omto.2020.04.007 PMC7200625 32382655

[216] Zhou W, Wang X, Rosenfeld MG. Histone H2A ubiquitination in transcriptional regulation and DNA damage repair. Int J Biochem Cell Biol. 2009;41(1):12–5. 10.1016/j.biocel.2008.09.016 18929679

[217] Williamson EA, Wray JW, Bansal P, Hromas R. Overview for the histone codes for DNA repair. Prog Mol Biol Transl Sci. 2012;110:207–27. 10.1016/B978-0-12-387665-2.00008-0 PMC4039077 22749147

[218] Dawson MA, Kouzarides T. Cancer epigenetics: from mechanism to therapy. Cell. 2012;150(1):12–27. 10.1016/j.cell.2012.06.013 22770212

[219] Di Nisio E, Lupo G, Licursi V, Negri R. The role of histone lysine methylation in the response of mammalian cells to ionizing radiation. Front Genet. 2021;12:639602. 10.3389/fgene.2021.639602 PMC8042281 33859667

[220] An S, Camarillo JM, Huang TY, Li D, Morris JA, Zoltek MA, et al. Histone tail analysis reveals H3K36me2 and H4K16ac as epigenetic signatures of diffuse intrinsic pontine glioma. J Exp Clin Cancer Res. 2020;39(1):261. 10.1186/s13046-020-01773-x PMC7687710 33239043

[221] Ahn HJ, Hwang SY, Nguyen NH, Lee IJ, Lee EJ, Seong J, et al. Radiation-induced CXCL12 upregulation via histone modification at the promoter in the tumor microenvironment of hepatocellular carcinoma. Mol Cells. 2019;42(7):530–45. 10.14348/molcells.2019.2280 PMC6681868 31362469

[222] Lee TG, Kim SY, Kim HR, Kim H, Kim CH. Radiation induces autophagy via histone H4 lysine 20 trimethylation in non-small cell lung cancer cells. Anticancer Res. 2020;40(5):2537–48. 10.21873/anticanres.14224 32366398

[223] Sharda A, Rashid M, Shah SG, Sharma AK, Singh SR, Gera P, et al. Elevated HDAC activity and altered histone phospho-acetylation confer acquired radio-resistant phenotype to breast cancer cells. Clin Epigenetics. 2020;12(1):4. 10.1186/s13148-019-0800-4 PMC6942324 31900196

[224] Zhang C, Guo C, Li Y, Ouyang L, Zhao Q, Liu K. The role of YTH domain containing 2 in epigenetic modification and immune infiltration of pan-cancer. J Cell Mol Med. 2021;25(18):8615–27. 10.1111/jcmm.16818 PMC8435423 34312987

[225] He JJ, Li Z, Rong ZX, Gao J, Mu Y, Guan YD, et al. m(6)A reader YTHDC2 promotes radiotherapy resistance of nasopharyngeal carcinoma via activating IGF1R/AKT/S6 signaling axis. Front Oncol. 2020;10:1166. 10.3389/fonc.2020.01166 PMC7411471 32850334

[226] Wu P, Fang X, Liu Y, Tang Y, Wang W, Li X, et al. N6-methyladenosine modification of circCUX1 confers radioresistance of hypopharyngeal squamous cell carcinoma through caspase1 pathway. Cell Death Dis. 2021;12(4):298. 10.1038/s41419-021-03558-2 PMC7979824 33741902

[227] Morel D, Jeffery D, Aspeslagh S, Almouzni G, Postel-Vinay S. Combining epigenetic drugs with other therapies for solid tumours - past lessons and future promise. Nat Rev Clin Oncol. 2020;17(2):91–107. 10.1038/s41571-019-0267-4 31570827

[228] Du Y, Zhou X. Targeting non-B-form DNA in living cells. Chem Rec. 2013;13(4):371–84. 10.1002/tcr.201300005 23828823

[229] Wang G, Vasquez KM. Dynamic alternative DNA structures in biology and disease. Nat Rev Genet. 2023;24(4):211–34. 10.1038/s41576-022-00539-9 PMC11634456 36316397

[230] Wolfe AL, Singh K, Zhong Y, Drewe P, Rajasekhar VK, Sanghvi VR, et al. RNA G-quadruplexes cause eIF4A-dependent oncogene translation in cancer. Nature. 2014;513(7516):65–70. 10.1038/nature13485 PMC4492470 25079319

[231] Ray U, Sharma S, Kapoor I, Kumari S, Gopalakrishnan V, Vartak SV, et al. G4 DNA present at human telomeric DNA contributes toward reduced sensitivity to gamma-radiation induced oxidative damage, but not bulky adduct formation. Int J Radiat Biol. 2021;97(9):1166–80. 10.1080/09553002.2021.1955997 34259614

[232] Kumari N, Vartak SV, Dahal S, Kumari S, Desai SS, Gopalakrishnan V, et al. G-quadruplex structures contribute to differential radiosensitivity of the human genome. iScience. 2019;21:288–307. 10.1016/j.isci.2019.10.033 PMC6838516 31678912

[233] Biffi G, Tannahill D, Miller J, Howat WJ, Balasubramanian S. Elevated levels of G-quadruplex formation in human stomach and liver cancer tissues. PLoS One. 2014;9(7):e102711. 10.1371/journal.pone.0102711 PMC4102534 25033211

[234] Fracchioni G, Vailati S, Grazioli M, Pirota V. Structural unfolding of G-quadruplexes: From small molecules to antisense strategies. Molecules. 2024;29(15):3488. 10.3390/molecules29153488 PMC11314335 39124893

[235] Mori K, Gotoh S, Yamashita T, Uozumi R, Kawabe Y, Tagami S, et al. The porphyrin TMPyP4 inhibits elongation during the noncanonical translation of the FTLD/ALS-associated GGGGCC repeat in the C9orf72 gene. J Biol Chem. 2021;297(4):101120. 10.1016/j.jbc.2021.101120 PMC8446798 34450161

[236] Berroyer A, Kim N. The functional consequences of eukaryotic topoisomerase 1 interaction with G-quadruplex DNA. Genes (Basel). 2020;11(2):193. 10.3390/genes11020193 PMC7073998 32059547

[237] Vlijm R, Mashaghi A, Bernard S, Modesti M, Dekker C. Experimental phase diagram of negatively supercoiled DNA measured by magnetic tweezers and fluorescence. Nanoscale 2015;7(7):3205–16. 10.1039/c4nr04332d 25615283

